# Nanomolar oligomerization and selective co-aggregation of α-synuclein pathogenic mutants revealed by single-molecule fluorescence

**DOI:** 10.1038/srep37630

**Published:** 2016-11-28

**Authors:** Emma Sierecki, Nichole Giles, Quill Bowden, Mark E. Polinkovsky, Janina Steinbeck, Nicholas Arrioti, Diya Rahman, Akshay Bhumkar, Philip R. Nicovich, Ian Ross, Robert G. Parton, Till Böcking, Yann Gambin

**Affiliations:** 1 EMBL Australia Node in Single Molecule Science, The University of New South Wales, Sydney NSW 2032 Australia; 2 Institute for Molecular Bioscience, The University of Queensland, St Lucia QLD 4072, Australia

## Abstract

Protein aggregation is a hallmark of many neurodegenerative diseases, notably Alzheimer’s and Parkinson’s disease. Parkinson’s disease is characterized by the presence of Lewy bodies, abnormal aggregates mainly composed of α-synuclein. Moreover, cases of familial Parkinson’s disease have been linked to mutations in α-synuclein. In this study, we compared the behavior of wild-type (WT) α-synuclein and five of its pathological mutants (A30P, E46K, H50Q, G51D and A53T). To this end, single-molecule fluorescence detection was coupled to cell-free protein expression to measure precisely the oligomerization of proteins without purification, denaturation or labelling steps. In these conditions, we could detect the formation of oligomeric and pre-fibrillar species at very short time scale and low micromolar concentrations. The pathogenic mutants surprisingly segregated into two classes: one group forming large aggregates and fibrils while the other tending to form mostly oligomers. Strikingly, co-expression experiments reveal that members from the different groups do not generally interact with each other, both at the fibril and monomer levels. Together, this data paints a completely different picture of α-synuclein aggregation, with two possible pathways leading to the development of fibrils.

α-Synuclein is a small 140 amino-acid long protein that plays an important role in the trafficking of synaptic vesicles and in the regulation of vesicle exocytosis, contributing to regulation of neurotransmitter release, synaptic function and plasticity^1^. α-Synuclein is very abundant in neurons, representing 1% of the cytosolic proteins. It is enriched at presynaptic terminals and mainly localizes in close proximity to, but not within synaptic vesicles^2^. How α-synuclein exactly controls trafficking is still unknown. Indeed, α-synuclein not only interacts with the synaptic vesicles, it can also interact with and potentially control proteins involved in the phosphatidyl acid metabolic pathway^3^ or act as a co-chaperone for the presynaptic SNARE complex^4^.

However, it is not the physiological role of α-synuclein that is the main subject of a wealth of literature, but rather its structure and aggregation propensity. Indeed, α-synuclein is one of the major components of the Lewy bodies^5^ associated with Parkinson’s disease (PD), multiple system atrophy (MSA), and other neurodegenerative disorders called synucleinopathies^6^. Mutations in the SNCA gene (encoding α-synuclein) were the first reported links between familial sporadic PD and perturbations at the molecular level^7^. It is widely accepted that aggregation of α-synuclein plays a pathological role. The exact “vector” of toxicity is however still ill-defined as aggregation of α-synuclein actually leads to a variety of aggregated forms. On route to the formation of frank neuronal inclusions, different soluble oligomers are formed^8^ that coalesce into proto-fibrils, before finally fibrillating and being incorporated into the inclusions^9^. The recent years have witnessed a paradigm shift and toxicity is now thought to arise from the formation of small soluble oligomers^10^,^11^, which form early in the aggregation pathway, rather than from the accumulation of mature fibrils. This hypothesis is still controversial though as long-term exposure to fibrils and inclusions may also prove to be neurotoxic through other mechanisms^12^,^13^. Interestingly, α-synuclein adopts a prion-like behavior when aggregating^14^,^15^. Auto-catalysis of aggregation has been demonstrated *in vitro*^16^,^17^ and more critically participates in the propagation of the phenotype in cell cultures^18^,^19^,^20^,^21^ and *in vivo*^22^,^23^. Different conformations of aggregates promote their own replication only, reminiscent of the prion strains^23^,^24^.

Dissecting the events that lead to the formation of fibrils is therefore essential to identify new therapeutic targets but can be experimentally challenging^17^. Most biochemical analyses of naturally aggregating proteins need to first overcome the hurdle of recombinant expression and purification/labelling. In most cases, the proteins of interest have to be rescued from insoluble fractions by forcing the protein into a non-oligomeric state using chemical or thermal denaturation. After this delicate step, one has to create *in vitro* conditions that would mimic their physiological aggregation. Using purified recombinant proteins, α-synuclein aggregation has been largely investigated but the results strongly depend on the experimental conditions^25^. A typical aggregation experiment requires high protein concentration and several days, and with factors such as buffer composition, temperature or agitation speed modifying the protein behavior^26^.

Since the self-assembly can proceed through multiple steps of dimerization, oligomerization and nucleation of fibrils, the ability to detect early and rare events in the kinetic process becomes crucial^27^. In this context, single-molecule fluorescence spectroscopy is especially attractive as they allow the detection of sub-populations in heterogeneous samples^27^,^28^.

Here, single-molecule fluorescence methods were coupled to cell-free protein expression to track oligomerization of the proteins in “real time” as the proteins are being expressed, in undisturbed samples. The aggregation study was performed directly in the cell-free translation reaction of the *Leishmania Tarentolae* Extracts (LTE)^29^,^30^ that possesses the full eukaryotic translation machinery. The use of genetically encoded fluorophores enabled a direct observation of the expressed proteins without purification, denaturation or labelling steps. This combination of methods also enabled to quantify concentration- and temperature-dependent aggregation, as well as co-aggregation between proteins tagged with different fluorophores. Applied to wild-type (WT) α-synuclein and five of its naturally occurring and pathogenic mutants (A30P^7^,^31^, E46K^32^, H50Q^33^,^34^, G51D^35^,^36^ and A53T^37^), the toolbox revealed an unexpected segregation of α-synuclein mutants into two different behaviors.

## Results

First, WT α-synuclein and 5 disease-associated α-synuclein mutants were expressed in LTE, with fluorescent proteins fused to their C-termini. A fast-folding and fast maturing version of monomeric GFP (“superfolder GFP”^38^ or sGFP for simplicity) was used to enable observation of the α-synuclein variants rapidly after their expression. The samples were observed after 2 h of expression at 27 °C, as the saturation of overall fluorescence signals the end of active protein production in LTE. The protein concentrations are calculated from the sGFP fluorescence. A calibration curve was obtained for *E. Coli* purified fluorescent proteins (sGFP and GFP-BBS9)^39^. Visualization under a confocal microscope showed homogeneous fluorescence for WT α-synuclein, but the presence of bright, small fluorescent specks diffusing in solution. In order to characterize further the possible oligomerization of α-synuclein, the samples were placed on a confocal microscope adapted to single-molecule fluorescence spectroscopy.

### Single-molecule detection of protein oligomerization and aggregation

**Figure 1:**
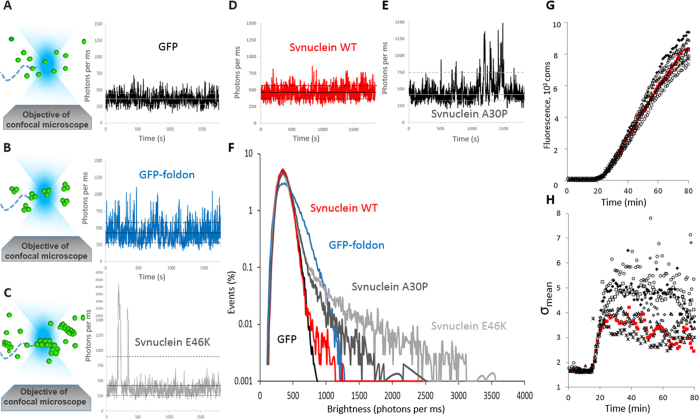
Brightness analysis is an ideal tool to monitor aggregation. (**A–C**) Schematic of single-molecule brightness experiment performed on a confocal microscope. Fluorescently-tagged proteins can freely diffuse in and out of the small detection volume, creating fluctuations in the intensity measured. (**A**) The fluorescence time trace for sGFP shows small statistical fluctuations around the average value. (**B**) The fluctuations are much larger when trimeric proteins are used (sGFP-foldon). (**C**) α-Synuclein E46K shows peaks corresponding to the diffusion of large aggregates containing multiple fluorophores. (**D**) No fluorescent bursts corresponding to fluorescent aggregates could be detected in the case of WT α-synuclein. (**E**) α-Synuclein A30P creates smaller objects, as indicated by the presence of fluorescent bursts of ∼1000 photons. (**F**) Analysis of distribution of intensity values measured in a 60 s time trace for sGFP, sGFP-foldon and the α-synuclein mutants clearly shows an increase of width of distribution upon oligomerization. The presence of a tail in the Gaussian distribution reflects the existence of bright events (aggregates) in the sample. (**G**) Time course of expression of WT α-synuclein and mutants (red squares: WT; crosses: GFP control; empty circles: E46K; grey triangles: A30P; black diamonds: H50Q; grey diamonds G51D; empty squares: A53T). The average fluorescence for each time trace is plotted as a function of time. (**H**) The variance of the distribution was calculated and plotted as a function of time. The data show extremely rapid oligomerization of the α-synuclein mutants E46K, A53T and H50Q that are the most prone to formation of large aggregates and fibrils.

Here, single-molecule detection of freely diffusing proteins is used, as illustrated in Fig. 1. On a confocal microscope, lasers are used to create a very small volume of illumination. Proteins are fluorescently tagged, but their fluorescence can only be excited and detected when they are present within the confocal volume. The illumination/detection “spot” is fixed spatially, but proteins constantly diffuse in and out of the confocal volume due to their Brownian motion.

In most single-molecule fluorescence measurements, the fluorescent proteins are diluted to extremely low concentrations (10–100 picomolar), to enable the detection of individual “bursts” of fluorescence caused by entry/exit of a single protein. Here the same single-molecule principles were applied to specifically detect the presence of oligomers and aggregates in a background of monomers, at much higher protein concentrations (up to 100 nanomolar) (see Fig. S1). Multiple proteins are constantly present in the detection volume, creating an average fluorescence, but the constant diffusion of the fluorophores causes fluctuations around this average intensity. Every entry of protein increases the fluorescence intensity detected in the confocal volume while every exit decreases the overall fluorescence. The presence of oligomers creates larger fluctuations as multiple fluorophores diffuse simultaneously; in this case the amplitude of the fluctuations increases linearly with the number of fluorophores present in the assemblies.

We first calibrated the amplitude of fluctuations using monomeric and trimeric sGFP, as shown in Fig. 1A and B. In the case of monomeric sGFP (Fig. 1A), the fluctuations of intensities corresponded well to the entry/exit of a single fluorophore in the detection volume. In the case of small oligomers such as trimers (sGFP-foldon), multiple fluorophores are diffusing simultaneously, creating fluctuations of larger amplitudes. We observe a broader spread of values for sGFP-foldon, with a distribution of intensities up to 3x larger than the ones observed for monomeric GFP (Fig. 1B). The α-synuclein mutants showed a very different behavior, with the presence of rare but very intense peaks, as shown for the E46K mutant in Fig. 1C. WT α-synuclein did not show much aggregation propensity as we only detected small fluctuations around the average value (Fig. 1D). It was immediately clear that several behaviors could be observed, as the maximum fluctuations observed for the A30P and G51D mutants were significantly smaller than the peaks observed for E46K, H50Q and A53T (see Fig. 1E and Fig. S2).

In this method, many molecules are interrogated within a short time-trace, enabling the detection of relatively rare aggregates. As the fluorescence intensity is measured in 1 millisecond time “bins”, a 60 seconds time-trace represents 60,000 fluorescence values. To take advantage of the large number of data, the distribution of fluorescence values can be plotted as histograms for detailed analysis (see Fig. S3). As shown in Fig. 1F, the distribution for a monomer (sGFP) is a Gaussian distribution, created by purely statistical fluctuations around the average value. The distribution for the controlled oligomer (sGFP-foldon) showed an increased overall width, reporting on the increased stoichiometry of the diffusing proteins. In the case of the α-synuclein mutants, the distribution was a composite of monomers and large oligomers creating a long tail of bright values (see Fig. S4). Based on the shape of the distributions, WT α-synuclein appears to be mainly monomeric (Fig. 1D). The distributions obtained for A30P and E46K are significantly different, as A30P tends to form small objects whereas E46K tends to create rare but very bright aggregates.

We next wanted to track oligomerization of α-synuclein as a function of time or as a function of expression levels. For screening purposes, we defined a simple parameter σ that quantifies the heterogeneity of the fluorescence time-trace in a concentration-independent manner: 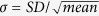 (where SD is the standard deviation and mean is the average fluorescence). The parameter σ reports precisely on the heterogeneity of the sample and its normalization enables an exact comparison between samples of different average fluorescence. This straightforward quantification is a simplified version of the “moment”^40^ or “Number and Brightness”^41^ analysis used in cell imaging to characterize the heterogeneity of distributions of fluorescence.

### Aggregation of the pathogenic α-synuclein mutants occurs co-translationally

To measure the kinetics of oligomerization, the expression of WT and mutants α-synuclein was started directly under the confocal microscope. 60 s time-traces were acquired continuously over the course of the reaction and the σ values were determined every minute (Fig. 1G,H)). To our surprise, as soon as GFP fluorescence is detected (typically within 20 minutes, Fig. 1G), σ reached its final value, suggesting that oligomerization occurs rapidly, almost co-translationally. The data showed two typical behaviors (Fig. 1H). The E46K, H50Q and A53T mutants reached similar, high sigma values (σ = 5) and they oligomerized significantly more than A30P and G51D (σ = 4) and WT α-synuclein. Interestingly even WT α-synuclein reached σ values significantly higher (σ = 3.5) than the control sGFP monomer.

### The pathogenic mutants of α-synuclein aggregate at low concentration

**Figure 2:**
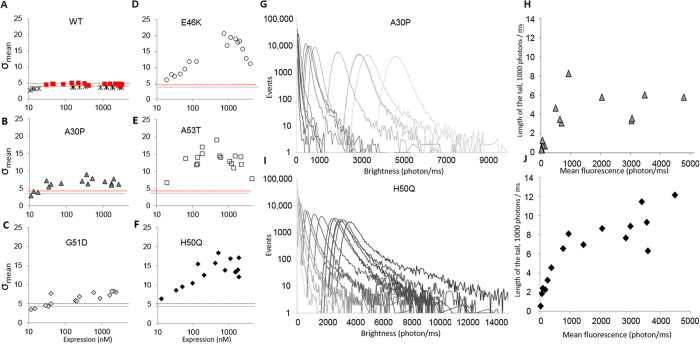
α-Synuclein mutants display two aggregation profiles. (**A–F**) Variance of the heterogeneity (σ) of the fluorescence time traces as a function of the final expression levels. WT α-synuclein (**A**) and mutants A30P (**B**), G51D (**C**), E46K (**D**), A53T (**E**) and H50Q (**F**) were expressed as C-terminal sGFP fusion proteins in LTE. The data show two behaviors, with the formation of either small oligomers (5 < σ <8) or large aggregates/fibrils (σ > 15). (**G**) Brightness profiles as a function of protein expression for A30P. Different DNA concentrations were used to prime the system, yielding a range of final expression levels. The traces were analyzed to obtain the distribution of intensity values (brightness, in counts per ms), allowing the direct observation of formation of large oligomers as a function of concentration. (**H**) Typical length of the tail of the distribution as a function of protein expression for A30P. The contribution of the large oligomers can be estimated by calculating the length of the tail of the distribution. (**I,J**) Brightness profiles (**I**) and length of the tail (**J**) as a function of protein expression for H50Q.

The difference of behavior between the mutants was investigated in more detail by performing titrations of the final expression levels. This was easily achieved by realizing a serial dilution of the encoding plasmid, leading to a gradual decrease of the efficiency of translation (Fig. S5). The results in Fig. 2A–F showed two striking features; (i) these simple experiments define critical concentrations where the aggregation saturates and reaches a plateau. Aggregation occurs at unexpectedly low concentrations, as maximal σ is reached at concentrations ranging between 10 and 100 nM. (ii) Again, two behaviors emerge clearly, with E46K, H50Q and A53T α-synuclein mutants aggregating significantly more than A30P and G51D.

The histograms of intensity values (Fig. 2G and I) reveal that the changes in σ values observed in Fig. 2B and F correlate with the apparition of rare but large oligomers, and are not due to the homogeneous formation of small, well-defined multimers. Indeed, as described above, homogeneous oligomerization is detected by a widening of the Gaussian core of the distribution (as in the case for sGFP-foldon, Fig. 1F) whereas rare, large events contribute to the creation of a tail in the distribution (Figs 1C–E and S4). E46K, H50Q and A53T were found to form more and more large objects (Fig. 2J) corresponding to assemblies of >100 proteins (based on the length of the tail distribution). In comparison, A30P (Fig. 2H) and G51D create smaller assemblies, encompassing ~30 proteins. Interestingly, the size of the oligomers formed does not seem to increase with protein concentration.

### Single-molecule TIRF imaging and ultracentrifugation confirm the size differences between A30P and E46K mutants

Both the brightness analysis and the σ parameter show a clear difference of behaviors between the mutants. However, like every other technique, different parameters such as the diffusion rate of the objects, intrinsic fluorescence properties of GFP, possibility of quenching/homo-FRET inside the oligomers/aggregates could affect quantitative measurements. In order to validate the oligomer sizes estimated above, two independent techniques, ultracentrifugation and single-molecule imaging were used. The cell-free extracts expressing WT α-synuclein, A30P and E46K mutants were spun at 80,000 rpm for 1h30 at 4 °C on top of a 10–60% sucrose gradient. 40 fractions were collected from each gradients and their overall fluorescence was measured, as shown in Fig. S6A. This clearly validated that the objects formed by E46K were larger than the oligomers of A30P. Further, single-molecule fluorescence imaging was used on samples fractionated by ultracentrifugation. Multiple fractions were tested on a single-molecule total internal reflection fluorescence (smTIRF) setup. In single-molecule TIRF, the proteins are adsorbed on the surface and the fluorescence is analyzed to “count” the number of fluorophores present in separate non-diffusing oligomers. Using WT α-synuclein, which was mostly monomeric, we calibrated the brightness of individual sGFP fluorophores. Multiple particles of A30P and E46K present in representative fractions were then measured and quantified. As shown in Fig. S6, we confirmed that A30P tends to form small oligomers gathering approximatively 30 proteins. The α-synuclein mutant E46K showed an additional population of brighter aggregates, made of 60 to >100 proteins.

### Electron Microscopy confirms the presence of fibrils

**Figure 3:**
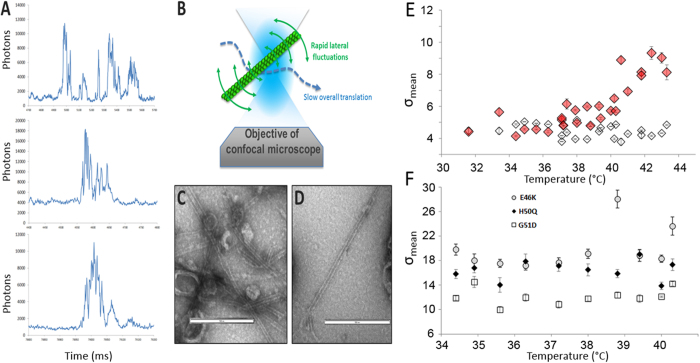
α-Synuclein mutants form fibrils. (**A**) Examples of single-molecule traces measured for the E46K mutant. As for H50Q and A53T, the large peaks of fluorescence have a specific profile, extremely jagged. This is compatible with the diffusion of a long object, such as a fibril. (**B**) Schematic of fibril diffusion into the confocal volume. The lateral movements can be more rapid than the displacement of the whole object; the core of the fibril would quickly “vibrate” in an out of the focal volume and create very rapid fluctuations of very large amplitude. (**C,D**) Formation of fibrils was confirmed by Electron Microscopy for E46K (**C**) and H50Q (**D**). Electron microscopy was performed on the LTE samples expressing WT and mutant synucleins, without purification. (**E**,**F**) Temperature denaturation of WT α-synuclein (**E**) and E46K, H50Q and G51D (**F**). WT and mutant α-synuclein were incubated at different temperatures for 30 minutes prior to fluorescence recording. 30 seconds time traces were analyzed and the average and standard deviation were obtained. σ is plotted as a function of temperature. Average ± SE for 5 different readings are presented. (**E**) α-Synuclein WT is destabilized above 40 °C. α-Synuclein tagged in C-terminal sGFP is presented in red and N-GFP WT α-synuclein is in white. (**F**) E46K (grey circles), H50Q (black diamonds) and G51D (white squares) aggregation is unaffected by temperature.

For the most aggregation-prone mutants, we observed a decrease in σ at high concentration (or in the kinetics experiments, see Fig. 1H and Fig. 2D–F), corresponding to the formation of large objects that slowly sediment at the bottom of the well. The shape of the fluorescence bursts informs on the morphology of the objects. The large peaks of fluorescence observed for E46K (Fig. 3A) have a specific profile, extremely jagged. This is compatible with the diffusion of an anisotropic object, such as a small fibril (Fig. 3B). Indeed, in the case of a fibril diffusing into the confocal volume, the lateral movements are actually more rapid than the displacement of the whole object. The core of the fibril quickly “vibrates” in an out of the focal volume and creates very rapid fluctuations of very large amplitude. Lysates expressing E46K (Fig. 3C) and H50Q (Fig. 3D) were observed under electron microscopy (EM), (see Methods). EM images confirmed the presence of fibrils in the sample and corroborated the single-molecule results. It also verified the absence of fibril in the WT α-synuclein sample (data not shown).

### WT α-synuclein aggregates above 40 °C

WT α-synuclein does not form aggregates in most conditions but thermal denaturation can be used to trigger aggregation. The LTE expressing WT α-synuclein and mutants were incubated at different temperatures for 30 minutes prior to single-molecule fluorescence acquisition. In most cases, the temperature had no effect on aggregation of the mutants and σ remained unchanged, as the proteins were already in oligomeric states (Fig. 3F). However, in the case of WT α-synuclein, an increase in σ could be detected at temperatures above 40 °C (Fig. 3E). When destabilized, WT aggregates in a similar way as A30P and G51D, as indicated by the low σ value (σ ⋍ 8–10).

### E46K, H50Q, A53T do not interact with WT, A30P or G51D

**Figure 4:**
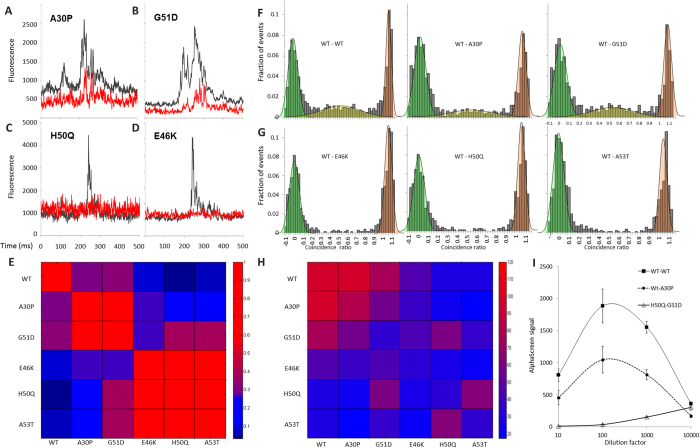
The two classes of mutants are mutually exclusive. (**A–D**) Representative fluorescence time traces of WT α-synuclein, tagged with sGFP at the C-terminus, in co-expression with A30P (**A**), G51D (**B**), H50Q (**C**) and E46K (**D**) expressed as C-terminal mCherry fusion proteins. Two overlapping lasers simultaneously excite the sGFP and mCherry fluorophores and the two emissions are recorded separately. Fluorescence trace for the WT is shown in red whereas fluorescence of the different mutants is shown in black. (**A,B**) Fluorescent bursts in the WT and mutants traces are coincident indicating that A30P (**A**) and G51D (**B**) can form co-fibrils with WT. (**C,D**) On the contrary, H50Q (**C**) and E46K (**D**) do not include WT into their fibrils. (**E**) Heatmap of co-aggregation between the different point-mutants of α-synuclein. Red indicates a strong co-aggregation whereas blue corresponds to no incorporation (see text for details). (**F,G**) Dual-color single-molecule coincidence experiments shows selective dimerization (see Methods). In F, the presence of a coincidence peak indicates that WT α-synuclein can form a dimer with itself A30P or G51D (left to right). The absence of population for 0.25 < C < 0.75 in G indicates that WT α-synuclein doesn’t interact with E46K, H50Q or A53T. (**H**) Heatmap of interaction between pair of proteins at the monomer level obtained by AlphaScreen. Red indicates to a strong interaction whereas blue corresponds to no interaction. (**I**) Representative AlphaScreen data obtained for three protein pairs: WT-WT (black squares, full line); WT-A30P (black squares, dotted line) and H50Q-G51D (open triangles). Serial dilutions of the samples were realized and the AlphaScreen signals were collected. The typical AlphaScreen curve has a bell shape, as illustrated with WT-WT or WT-A30P whereas a flat line at background level is obtained in the absence of interaction (H50Q-G51D). The maximum signal was used to calculate the binding index in H. Data correspond to average ± SE for 3 different experiments.

The striking difference of behavior between mutants led us to investigate their ability to interact and co-aggregate. We were especially interested to see whether WT α-synuclein aggregation could be triggered by “seeds”, fibril formed by the α-synuclein mutants. To maximize the probability of co-aggregation, WT α-synuclein tagged with a C-terminal sGFP and the mutants tagged with a C-terminal monomeric Cherry (mCherry) were co-expressed and two-color single-molecule coincidence experiments were performed. In this experiment, two lasers, one exciting sGFP and the other exciting mCherry, are focused in the same confocal volume. Here the method focuses on detecting the co-diffusion of sGFP and mCherry in the bright bursts, signaling that oligomers diffuse in and out of the two-color detection volume (see Fig. S7 for details). As illustrated in Fig. 4, WT α-synuclein was recruited into the fibrils formed by A30P and G51D but was completely excluded from the larger fibrils formed by E46K, H50Q or A53T. To investigate further this surprising result, we performed systematically co-aggregation experiments between all α-synuclein pairs. The results, presented in the heatmap of Fig. 4E, revealed that not only WT α-synuclein, but A30P and G51D also, are excluded from the fibrils formed by the 3 other mutants. However, E46K, H50Q and A53T are able to co-aggregate. Similarly, A30P, G51D and WT can be recruited into each other fibrils.

The absence of co-aggregation doesn’t necessarily imply that proteins do not interact, especially if there is a change of conformation between the monomeric state and the aggregated state. To verify if the segregation observed during aggregation was true at the monomer level, we performed two-color single-molecule analysis, diluting the sample to picomolar concentrations. In these conditions, the small diffusing objects corresponding to monomers or dimers can be observed individually. Figure 4F,G show that WT α-synuclein is only able to interact with itself, G51D and A30P but not with E46K, H50Q or A53T. To verify these results and widen the test to interactions between all protein pairs, a proximity assay was used^39^ (Amplified Luminescent Proximity Homogeneous Assay Screen, or AlphaScreen). The AlphaScreen assay utilizes two nanobeads to detect protein-protein interactions with high sensitivity (See Fig. S8). Protein pairs are co-expressed and their sGFP/mCherry fluorophores are captured on the two different nanobeads. A luminescence signal is detected if the two different proteins bring the two nanobeads in close proximity. The AlphaScreen results, presented in Fig. 4H in a heatmap format, show the same tendencies as the one in Fig. 4E, with WT α-synuclein interacting with A30P and G51D. AlphaScreen data also reveal that A30P can dimerize and interact with G51D, though with low affinity. Finally, no interactions at the monomer level were detected between WT α-synuclein and the other mutants or between the different mutants. Also, we found no signs of dimerization of E46K, H50Q or A53T, indicating that the dimers/small oligomers of those mutants are absent or quickly form larger oligomers or aggregates.

## Discussion

### Oligomers and fibrils

Our cell-free expressed proteins were found to form oligomers and small aggregates at concentration around 10–100 nM and in less than 20 minutes. Cell-free expression occurs at 27 °C and the fluorescence experiments are realized at room temperature. A number of similar studies^42^,^43^,^44^,^45^,^46^ have been reported using purified recombinant proteins. Typically, WT α-synuclein fibrils, as detected by ThT binding assays, are obtained by incubating monomeric WT α-synuclein at high micromolar concentrations (50–1000 μM) at 37 °C, upon agitation for 1–5 days^42^,^43^,^46^. Fibrils from α-synuclein mutants are formed in almost the same conditions, with a 2–3 fold increase or decrease in maturation time. Our results differ from these reports by 2–3 orders of magnitude in terms of concentration and time, leading us to believe that we observed the formation of the first oligomers and pre-fibrillar species at the initiation of the aggregation process.

We first wanted to rule out the possibility that the behaviors observed were created or affected by the genetically encoded fluorophores or resulting from the expression in the cell-free system. First, we performed multiple experiments to interrogate the influence of the GFP tags. These tags are larger than α-synuclein itself and could interfere with its natural ability to aggregate. Note that one would expect the tag to block aggregation, and not enhance polymerization. Here C-terminal tagging was used as α-synuclein is believed to acquire structure in its N-terminus, while keeping its C-terminus tail flexible. Indeed, the first tests performed showed that N-terminal tagging of WT and mutants α-synuclein significantly decreased the oligomerization. Most of the data were then conducted on C-term tagged α-synuclein. A closer look at the initial screen showed that N-terminal tagging blocked completely the aggregation of WT α-synuclein, A30P and G51D mutants, but oligomerization of E46K and H50Q was still possible (see Fig. S9). Again, this suggests a fundamental difference between WT α-synuclein and the mutants E46K, H50Q and A53T.

Two different fluorophores were used, monomeric sGFP and monomeric mCherry, belonging to different families of fluorescent proteins. These two fluorophores gave exactly the same aggregation pattern for each mutant, suggesting that the aggregation is not driven or enhanced by the presence of the fluorophore. These fluorophores are extensively used in fluorescence microscopy and are unlikely to create aggregates at nanomolar concentrations, as observed here (Fig. 2).

To further validate this hypothesis, the WT and mutant α-synuclein constructs were cloned as C-terminal His-tagged fusions and labeled post-expression with small organic dyes. A DyLight488-fluorescent derivative of Tris-NTA was synthetized according to ref. 47. As shown in Fig. S10, the Tris-NTA recognizes the 6-His tag with nanomolar affinity, enabling efficient labelling of the proteins post-translation and post-aggregation. The Tris-NTA compound was added in equimolar ratio with untagged WT and mutant α-synuclein after 2 h of expression in LTE, incubated for 30 minutes and single-molecule fluorescence traces were acquired. The data presented in Fig. S10 showed very similar profiles to the ones obtained with sGFP-tagged proteins.

We next investigated whether elements present in the LTE could be responsible for the enhanced oligomerization observed in our experiments. The cell-free extracts are carefully spun multiple times to remove membrane debris and organelles after cell lysis, but some manufactured batches display higher lipid content. This is useful in other projects, where small transmembrane proteins or amphiphilic peptides can be expressed, and where membrane-driven assembly is observed. Batches of cell-free lysates with opposite properties and various manufacturing protocols were tested. The same concentration-dependent aggregation was observed in all batches, as shown in Fig. S11. The influence of the different elements of the lysates was then evaluated. To be translation-competent, the cell extracts have to be supplemented with various components such as amino-acids for protein expression, NTPs (nucleotides triphosphates) for energy, cryo-protectants, etc. The aggregation propensity of WT α-synuclein, A30P and E46K mutants was tested under different conditions of supplementation, notably varying the amount of ATP, PEG/spermidine (cryo-protectant and crowding agents), oligonucleotides present in the mixture (Fig. S12). We observed no significant difference between the different conditions tested showing that none of these elements was responsible for the behaviors we observed. A possible explanation for fast oligomerization in our system compared to pure buffer conditions is the difference in molecular crowding. As the cell-free extracts contain a high concentration of proteins, they provide a crowded environment even in the absence of PEG and could accelerate the rate of oligomerisation. It was shown that IDPs tend to aggregate more in crowded environments, irrespective of the nature of the protein and the crowding agent^48^. The common effect of BSA, lysozyme or different polymers suggests that oligomerization is enhanced by effect of excluded volume, and not due to interactions or conformational changes^49^,^50^.

Overall, the fact that WT α-synuclein does not aggregate readily, the differences observed between mutants, and the exquisite selectivity of co-aggregation all suggest that the tags or the cell-free expression system are not responsible for the aggregation of the α-synuclein mutants, and that our experiments captured intrinsic propensities to spontaneously self-assemble.

Another possibility is that the objects we detect are not the fully-formed fibrils described in the literature. As the protein concentrations used here are lower than used for most aggregation experiments, the oligomerization is probably favored and the differences between WT and mutants are more striking. In one of the first paper published after the discovery of the A30P and A53T mutations, Uversky and Fink showed that the oligomerization occurred at much lower concentrations for A30P and A53T compared to WT^51^. Changes were detected as low as 6 micromolar, while WT remained perfectly monomeric. In our experiments, in a low micromolar concentration range, we believe that the ~30-mers formed by A30P and G51D (or WT upon thermal denaturation) would qualify as oligomers rather than fibrils. Indeed, different studies^52^,^53^ have reported the presence of oligomers containing around 30 proteins in samples of WT and mutant α-synuclein. Chen *et al*. report on the presence of 3 types of oligomers in their preparation of α-synuclein, with fractions containing small oligomers (18 ± 7 monomers/object), large oligomers (29 ± 10 monomers/object) and fibrils containing more than 90 monomers^53^. The latter objects could correspond to the oligomers of E46K, H50Q or A53T that we detect. Starting from purified recombinant α-synuclein, production of oligomeric species of α-synuclein is faster, with oligomers being obtained for example by incubation of low millimolar solution at either room temperature for 24 h^28^,^54^ or 5 h at 37 °C^52^,^55^. Oligomerization was actually detected in the first 20 minutes after solubilization of freeze-thawed monomeric α-synuclein^52^. Interestingly, immediately after mixing proteins labelled separately with A488 and A594, Tosatto *et al*. are detecting coincidence and FRET in oligomers of synuclein mutants, but not in synuclein WT^56^.

Our data also correlate with results obtained in cells using number and brightness analysis^57^. Overexpressed α-synuclein-GFP was found to oligomerize in SH-SY5Y cells above a threshold concentration of 90 nM, which is similar to the threshold concentrations we detected for the mutant α-synuclein. WT α-synuclein created oligomers containing up to 10 monomers before being sequestered by lysosomes. This feature can explain why no bigger objects could be identified in this study and does not rule out the possibility that the first stable intermediates are oligomers containing around 30 monomers.

A30P and G51D may mainly form oligomers but E46K, H50Q and A53T form larger fibrils as validated by EM. In our study, we investigate the protein a short time after translation, or even while it is translated. Therefore we believe we mainly observe the first steps of the aggregation process and can detect the initial oligomers or pre-fibrillar species that will ultimately lead to the formation of the described fibrils (Fig. S13 for longer kinetics). As our system only detects freely diffusing aggregates, it is possible that larger fibrils would sediment and that their contribution is under-estimated at later time points, and we focused our analysis on the early stages of oligomerization.

### Two classes of mutants

We identified two groups of behavior. In our assay, E46K, H50Q and A53T rapidly form large objects whereas A30P, G51D and WT tend to aggregate less and form smaller objects. This is in good agreement with the literature. Many studies showed that E46K^58^, H50Q^59^,^60^ and A53T^51^,^61^,^62^ aggregate more and more rapidly than WT α-synuclein *in vitro* using recombinant proteins. More fibrils could also be detected in cells^63^,^64^. A30P^61^,^62^ and G51D^65^,^66^ were found to aggregate to the same or a lesser extent than WT α-synuclein *in vitro* and form the same number of fibrils in cells. A recent study showed that the types of oligomers formed by A30P and A53T are different at the end of the lag phase^56^.

Our most striking finding is the fact that WT α-synuclein, A30P and G51D could not be recruited into the rapidly formed fibrils of E46K, H50Q or A53T. Yet A30P, G51D and WT α-synuclein to a lesser extent are able to form their own fibrils and importantly incorporate each other into these objects. This suggests that E46K, H50Q and A53T on one hand and A30P, G51D and WT α-synuclein on the other hand, can give rise to two distinct types of fibrils. It has already been shown that E46K and A53T formed different fibrils compared to WT α-synuclein^67^,^68^,^69^ whereas fibrils of A30P have a similar morphology compared to WT fibrils^69^,^70^. At the cellular level, differences in fibril morphologies can profoundly affect the biological effect^24^. Furthermore, the absence of co-aggregation between the two families is reminiscent of the “strains” in prion disease. It was observed that synthetic WT α-synuclein can assemble into two different strains of fibrils, with different conformations and different effects in neurons^71^. The difference of morphology between the oligomers of A30P and the small aggregates of E46K is suggested by the EM images (Fig. S14) and could be detected by FRET analysis (Fig. S15). When analyzing the oligomers of α-synuclein A30P, obtained from the co-expression of the C-terminally tagged mCherry and sGFP proteins, we observed a modest FRET between the GFP and Cherry fluorophores of 0.2. The same analysis for E46K aggregates shows no FRET peak. As FRET analysis report on the distance between two fluorophores, we concluded that the spatial arrangement of the proteins is different between the two mutants. These results are similar to previous reports^56^,^72^,^73^.

As mutations in *SNCA* are mainly heterozygous, the easier incorporation of WT α-synuclein into aggregates of A30P and G51D could be relevant to the pathophysiologies associated with these mutations. The difference of behavior between H50Q and G51D is particularly notable. Although the single-point mutations are next to each other, the two mutants belong to different categories and do not associate. Previous comparison^66^ also showed that H50Q and G51D have different characteristics: H50Q was aggregating faster than WT α-synuclein *in vitro* whereas G51D formed fewer fibrils at a slower rate^74^. Contrary to H50Q, oligomers of G51D displayed less membrane permeabilization ability^75^ yet G51D is much more toxic than H50Q in cells^36^. The disease caused by G51D mutation also has distinct clinical and neuropathological features compared to the pathology associated with H50Q^76^, with an earlier onset and a more rapid progression^77^ as well as differences in the localization and composition of the inclusions. We now propose that part of the differences between the two pathologies may arise from the segregation of WT α-synuclein at an earlier stage in the disease caused by G51D compared to H50Q.

### A new model for α-synuclein aggregation

The absence of interaction between the two groups at the monomer level suggests that the fate of the protein is actually encoded in the mutation. Indeed, if the model of an initial perturbation of the native fold was true, a protein, especially WT α-synuclein, should be able to sample the different configurations leading to different aggregates/fibrils. In this case, dimers or small oligomers of mixed species should be detected. However, we observe that WT α-synuclein, A30P and G51D are never able to interact with E46K, H50Q or A53T, by AlphaScreen and two-color single-molecule coincidence.

**Figure 5:**
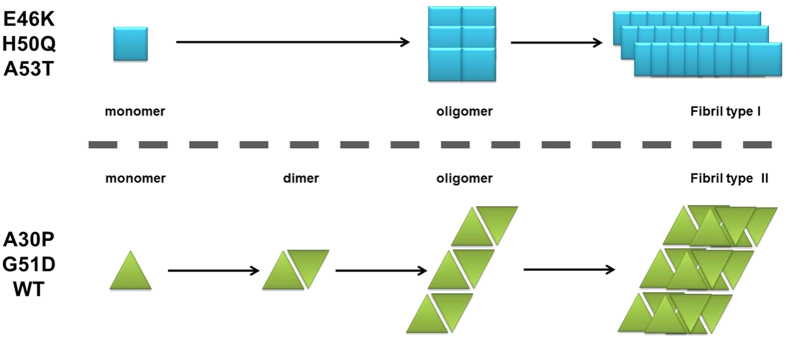
Proposed model of mutually exclusive aggregation paths. In this model, two types of oligomers and fibrils are formed. Mutations E46K, H50Q or A53T prime α-synuclein in a conformation that leads to fast oligomerization, without formation of dimers. Indeed, we were unable to detect dimerization or interaction in this group by AlphaScreen. The kinetic analysis also shows that the first oligomers formed are larger, as indicated by a higher σ parameter. These small objects can rapidly evolve into type I fibrils. A30P and G51D resemble a destabilized WT α-synuclein. The protein has a tendency to dimerize, as detected by AlphaScreen, single-molecule coincidence and brightness analysis. Formation of oligomers of 30 or so proteins follows, and more seldom leads to formation of type II fibrils.

Based on these observations, we propose a new model of aggregation depicted in Fig. 5. In this model, two paths are available and mutually exclusive. Proteins are engaged in either path from their synthesis. Mutations E46K, H50Q or A53T prime α-synuclein for fast oligomerization. We were unable to detect dimerization or interaction in this group by AlphaScreen and single-molecule coincidence, suggesting that the formation of the dimer is either transient or unfavored. Previous reports suggested that the residues 43 to 60 α-synuclein are part of the dimerization interface^78^ so it is possible that mutations E46K, H50Q or A53T perturb this process. The kinetic analysis also shows that the first oligomers formed are larger, as indicated by a higher σ parameter. These small objects can rapidly evolve into type I fibrils. A30P and G51D resemble a destabilized WT α-synuclein^79^,^80^. The protein has a tendency to dimerize, as detected by AlphaScreen, two-color single-molecule coincidence and brightness analysis. This dimerization propensity has been noted before^81^,^82^. Formation of oligomers of ~30 proteins follows, that more rarely leads to formation of type II fibrils.

## Conclusion

The identification of two distinct classes of mutants is primarily supported by the co-aggregation and interaction data not only of the mutants with WT α-synuclein, but between the mutants themselves. It is the systematic analysis of the 6 proteins that lead us to hypothesize that there was a fundamental distinction between the classes. This idea prompted us to propose a new model for α-synuclein aggregation that will hopefully be amended by new observations.

It will now be interesting to test whether the different aggregates formed by the mutants relate to the different species formed along the aggregation pathway of WT α-synuclein. This could provide new models for the development of new therapeutics, particularly in PD and MSA.

## Methods

### Preparation of LTE

*Leishmania tarentolae* cell-free lysate was produced as described by Johnston & Alexandrov^29^,^30^,^83^. Briefly, *Leishmania tarentolae* Parrot strain was obtained as LEXSY host P10 from Jena Bioscience GmbH, Jena, Germany and cultured in TBGG medium containing 0.2% v/v Penicillin/Streptomycin (Life Technologies) and 0.05% w/v Hemin (MP Biomedical). Cells were harvested by centrifugation at 2500 x g, washed twice by resuspension in 45 mM HEPES, pH 7.6, containing 250 mM Sucrose, 100 mM Potassium Acetate and 3 mM Magnesium Acetate and resuspended to 0.25 g cells/g suspension. Cells were placed in a cell disruption vessel (Parr Instruments, USA) and incubated under 7000 KPa nitrogen for 45 minutes, then lysed by rapid release of pressure. The lysate was clarified by sequential centrifugation at 10 000 x g and 30 000 x g and anti-splice leader DNA leader oligonucleotide was added to 10 μM. The lysate was then desalted into 45 mM HEPES, pH 7.6, containing, 100 mM Potassium Acetate and 3 mM Magnesium Acetate, supplemented with a coupled translation/transcription feeding solution and snap-frozen until required.

### Gateway plasmids for cell-free protein expression

The proteins were cloned into the following cell free expression Gateway destination vectors: N-terminal GFP tagged (pCellFree_G03), C-terminal sGFP tagged (pCellFree_G04) or C-terminal mCherry-cMyc tagged (pCellFree_G08)^84^. The Open Reading Frames corresponding to WT and mutants α-synuclein were synthesized (IDT). Transfer of ORFs between vectors was carried using Gateway PCR cloning protocol based on insert amplification with primers to attB1 and attB2 sites (Forward primer: GGGGACAAGTTTGTACAAAAAAGCAGGCTT (nnn) 18–25, Reverse primer: (nnnn) 18–25 AACCCAGCTTTCTTGTACAAAGTGGTCCCC)^85^. For the Tris-NTA experiments, ORFs were transferred to a C-terminal 6-His tag vector.

### Single-molecule fluorescence spectroscopy

Single molecule spectroscopy was performed as described previously^39^,^86^. Proteins were expressed in LTE and then diluted in buffer A (25 mM HEPES, 50 mM NaCl). A volume of 20 μL of each sample was placed into a custom-made 192-well silicone plate with a 70 × 80 mm glass coverslip (ProSciTech). Plates were analyzed at room temperature on a Zeiss Axio Observer microscope with a custom-built data acquisition setup.

We performed analysis in two different ranges of concentration. To study the interaction between WT and mutant α-synuclein at the monomer level, the proteins were diluted to 10–100 picomolar concentrations. This enables the individual detection of single proteins but the extreme dilution required by these measurements has a few disadvantages. Protein complexes can dissociate into monomers, or stick non-specifically to the surfaces of the measurement chambers. A typical single-molecule measurement samples a few hundreds of individual proteins within 10 minutes, and rare oligomers or aggregates can be difficult to characterize. In order to gain a better picture of the oligomerization and aggregation of the proteins, we acquired time-traces at higher concentrations, between 1 and 100 nanomolar.

### Analysis of aggregation

For intensity measurements, C-terminal sGFP-labeled proteins were expressed in 10 μL of LTE using 20 nM of DNA template and incubated for 2 h at 27 °C. A 488 nm laser beam was focused in the sample volume using a 40x/1.2 NA water immersion objective (Zeiss). The fluorescence of GFP was measured through a 525/20 nm band pass filter, and the number of photons collected in 1 ms time bins (*I*(*t*)) was recorded. The proteins were diluted 10 times in buffer A to obtain the traces shown in Fig. 1 and Fig. S1.

The fluorescent time-trace I(t) obtained shows the presence of intense bursts of fluorescence for the mutants, with values well over the typical fluctuations of I(t). The presence of these bursts increases the standard deviation of the distribution. To compare the aggregation at different concentrations, we used the sigma parameter which is the standard deviation normalized by the square root of the mean signal.


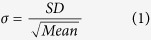


### Aggregation as a function of expression levels

To obtain different final concentrations of the proteins, a serial dilution of the plasmid encoding WT and mutants α-synuclein tagged with a C-terminal sGFP was realized (Fig. S5). Starting from a 100 nM stock, 16 steps of 1.25× dilutions were obtained. 1 μL of the diluted DNA was used to prime 9 μL of LTE. Expression was carried out at 27 °C for 3 h before the samples were diluted 11 times and submitted to aggregation analysis (45 seconds time traces were acquired).

### Time course of aggregation

30 μL of lysate were introduced in a well under the microscope. Expression was started directly under the microscope by addition of the plasmid at a 10 nM final concentration. The well was sealed by applying a glass coverslip to limit evaporation. Time traces of 60 seconds were obtained every minute for up to 3 h. Aggregation was analyzed as described previously.

### Single-molecule TIRF imaging of α-synuclein aggregates

Fusion proteins of α-synuclein with sGFP were expressed as described above and aggregates were enriched from the cell lysate using gradient centrifugation. Glass coverslips (#1.5 Warner Instruments) were cleaned with 70% (v/v) ethanol, dried and treated in a plasma cleaner (Harrick Plasma). Aggregates were absorbed from solution onto freshly cleaned coverslips. Coverslips were then rinsed with imaging buffer to remove unbound protein and imaged using an inverted TIRF microscope (TILL Photonics) with Zeiss α Plan-apochromat 100× oil objective (1.46 NA), 488 nm laser line and electron-multiplying charge-coupled device camera (Andor). Spots in the fluorescence image were detected as local maxima and fluorescence intensities were extracted by point-spread function fitting using image analysis software implemented using MATLAB.

### Electron Microscopy

WT and mutants α-synuclein expressing cell-free extracts were absorbed onto glow-discharged carbon coated formvar grids and stained with 1% uranyl acetate (UA) solution. Similar results were observed for proteins adsorbed on poly-L-lysine coated formvar grids. After washing, sample was further contrasted by 1% UA and imaged in an JEOL1011 electron microscope at 80 kV equipped with a Morada Soft Imaging System 4 K × 4 K camera at twofold binning under the control of iTEM (Olympus, Japan).

### Aggregation as a function of temperature

Plasmids encoding all proteins tagged with C-terminal GFP were added to 60 μL of lysate to a final concentration of 17 nM. Expression was carried out at 27 °C for 2 h30. The reaction mix was then split in 12 samples of 5 μL each which were submitted to a gradient of temperature using an Eppendorf Mastercycler Gradient PCR machine. All proteins were subjected to a gradient of (37 ± 3) °C for 30 minutes. Additionally, WT α-synuclein was also submitted to a gradient of (40 ± 3) °C for 30 minutes. The samples were then diluted 11 times and subjected to the aggregation analysis (5 times traces of 10 seconds were acquired for each sample).

### Co-aggregation measurements

Plasmids for WT α-synuclein and mutants C-terminally tagged with sGFP (4 nM) and mCherry (19 nM) were introduced in 6 μL lysate and co-expressed for 3 h 30 at 27 °C. The samples were diluted 11 times and fluorescence time traces were acquired for 60 seconds. Two lasers (488 nm and 561 nm) were focused in solution using a 40×/1.2 NA water immersion objective (Zeiss). Fluorescence was collected and separated using a 565 nm dichroic mirror; signal from sGFP (I_G_(t)) was passed through a 525/20 nm band pass filter, while fluorescence from mCherry (I_C_(t)) was filtered by a 580 nm long pass filter. The fluorescence of the two channels was recorded simultaneously in 1 ms time bins. Average and standard deviation for the distribution of fluorescence in the Cherry channel were calculated. For each event above average +3 SD, the ratio R was calculated as


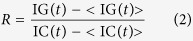


where I_C_(t) is the intensity in the Cherry channel, <I_C_(t)> is the average intensity in the Cherry channel, I_G_(t) is the intensity in the GFP channel and <I_G_(t)> is the average intensity in the GFP channel (see Fig. S7 for more details). The averaged R for more than 100 events was used to create the heatmap in Fig. 4E.

### Single molecule coincidence measurements

For coincidence experiments, C-terminal sGFP-labeled and C-terminal mCherry-Myc labelled proteins were co-expressed using 20 and 40 nM of DNA template respectively, in 10 μL of LTE for 3 h at 27 °C. Two lasers (488 nm and 561 nm) were focused in solution using a 40×/1.2 NA water immersion objective (Zeiss). Fluorescence was collected and separated using a 565 nm dichroic mirror; signal from GFP (I_G_(t)) was passed through a 525/20 nm band pass filter, while fluorescence from Cherry (I_C_(t)) was filtered by a 580 nm long pass filter. The fluorescence of the two channels was recorded simultaneously in 1 ms time bins. The threshold for positive events was set at 80 photons/time bin. For each event, the intensities of the GFP and Cherry bursts were corrected for background and leakage (6% leakage of the GFP intensity into the Cherry channel). The coincidence C was then measured as the corrected Cherry signal (I_C_), divided by the total intensity of the burst (C = I_C_/[I_G_ + I_C_]). In the absence of Cherry fluorescence, C is close to zero, while in the absence of GFP, C tends towards 1. Events with 0.25 < C < 0.75 are considered coincident events. The number of events for each ratio C was counted and normalized to the total number of events to give a probability P(C). Histograms of single-molecule coincidence (P(C) as a function of C) were obtained by measuring >1,000 events per interaction, and fitted by Gaussian peaks for GFP-only, coincidence and Cherry-only contributions. The bound fraction was calculated as the proportion of coincidence (0.25 < C < 0.75) to total events.

### AlphaScreen Assay

AlphaScreen relies on a system of two nanobeads^87^,^88^. The donor bead, which in our system couples with GFP-tagged protein, can be excited at 650 nm and emit singulet oxygens. The acceptor bead, which is coated with anti-myc antibodies, contains phtalocyanate, which is excited by singulet oxygen and emits luminescence. Because the singulet oxygen has a limited lifetime, detection of luminescence only occurs if the beads are in proximity. This in turn can only happen if the proteins coating the beads are interacting and keeping the beads next to each other. We in general use this assay for its high sensitivity that detects low affinity interactions^89^.

The AlphaScreen Assay was performed using the cMyc detection kit and Proxiplate-384 Plus plates (PerkinElmer) as described previously^39^. Every pair of proteins (one protein tagged with C-terminal sGFP and the other tagged with C-terminal mCherry-cMyc) was co-expressed using 20 and 40 nM of DNA template respectively, in 10 μL of LTE for 3 h at 27 °C.

The LTE lysate co-expressing the proteins of interest was diluted in buffer A (25 mM HEPES, 50 mM NaCl).

A four-fold serial dilution of each sample was realized to find the optimal loading of the beads. At high dilution (low concentration), the beads are not correctly coated with proteins and the signal is low. At low dilution (high concentration), proteins in solution can compete for interactions with the beads-bound proteins and the signal decreases again.

For the assay, 12.5 μL (0.4 μg) of Anti-cMyc coated Acceptor Beads in buffer B (25 mM HEPES, 50 mM NaCl, 0.001% NP40, 0.001% casein) were aliquoted into each well. This was followed by the addition of 2 μL of diluted sample and 2 μL of biotin labeled GFP-Nanotrap^90^ in buffer A. The plate was incubated for 45 minutes at RT. Afterward, 2 μL (0.4 μg) of Streptavidin coated Donor Beads diluted in buffer A, were added, followed by incubation in the dark for 45 minutes at RT. The AlphaScreen signal was recorded on an Envision Multilabel Plate Reader (PerkinElmer), using the manufacturer’s recommended settings (excitation: 680/30 nm for 0.18 s, emission: 570/100 nm after 37 ms). The resulting bell-shaped curve is an indication of a positive interaction, while a flat line reflects a lack of interaction between the proteins (Suppl. Fig. S8). The measurement of each protein pair was repeated a minimum of three times using separate plates.

A Binding Index (BI) was calculated for each pair of proteins using the following formula:


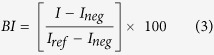


For each experiment, I is the highest signal level (top of the hook effect curve) and I_neg_ is the lowest (background) signal level. The signals are then normalized to the I_ref_ signal obtained for the interaction of WT α-synuclein with itself. The resulting data are then averaged among the different experiments.

## Additional Information

**How to cite this article**: Sierecki, E. *et al*. Nanomolar oligomerization and selective co-aggregation of α-synuclein pathogenic mutants revealed by single-molecule fluorescence. *Sci. Rep.* **6**, 37630; doi: 10.1038/srep37630 (2016).

**Publisher's note:** Springer Nature remains neutral with regard to jurisdictional claims in published maps and institutional affiliations.

## Supplementary Material

Supplementary Information
