# Supplementary material for: Nanomolar oligomerization and selective co-aggregation of α-synuclein pathogenic mutants revealed by single-molecule fluorescence
Source: Sci Rep. 2016 Nov 28;6:37630. doi: 10.1038/srep37630 (PMC5385372; doi:10.1038/srep37630)
Supplement: Supplementary Information [file srep37630-s1.pdf]

ADDITIONAL FILES

Nanomolar oligomerization and selective co-aggregation of α-synuclein pathogenic mutants revealed by single-molecule fluorescence

Emma Sierecki[+1], Nichole Giles[1], Quill Bowden[1], Mark Polinkovsky[1], Janina Steinbeck[2], Nicholas Arrioti[2], Diya Rahman[2], Akshay Bhumkar[1], Philip Nicovich[1], Ian Ross[2], Robert Parton[2], Till Boecking[1], Yann Gambin[+1].

[1]*EMBL Australia Node in Single Molecule Science, The University of New South Wales, Sydney NSW 2032 Australia*

[2] *Institute for Molecular Bioscience, The University of Queensland, St Lucia QLD 4072, Australia*

[+] Correspondence should be addressed to: e.sierecki@unsw.edu.au or y.gambin@unsw.edu.au

Figure S1 Principle of the single-molecule detection of oligomers and aggregates.

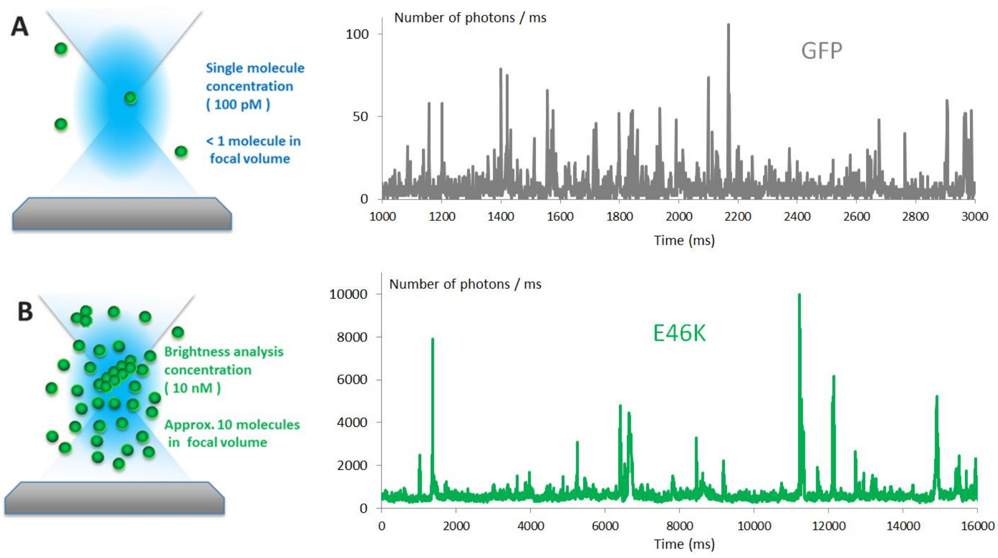

(A) Conditions for detection of individual proteins in a typical single-molecule fluorescence experiment. As shown on the left, proteins are extremely diluted so that the probability of observing two different proteins at the same time is almost zero. The fluorescence time-trace (right) displays short bursts of fluorescence, here as monomeric GFPs diffuse in and out of the focal volume.

(B) Conditions used in this study to quantify the oligomerization propensity of WT α-synuclein and α-synuclein mutants. The samples are more concentrated, so that multiple proteins are constantly present in the focal volume. Here the fluorescence time-traces show large bursts of fluorescence as oligomers carry many fluorophores simultaneously in and out of the detection volume.

Figure S2- The fluorescence traces measured for the different synuclein mutants show different aggregation propensity.

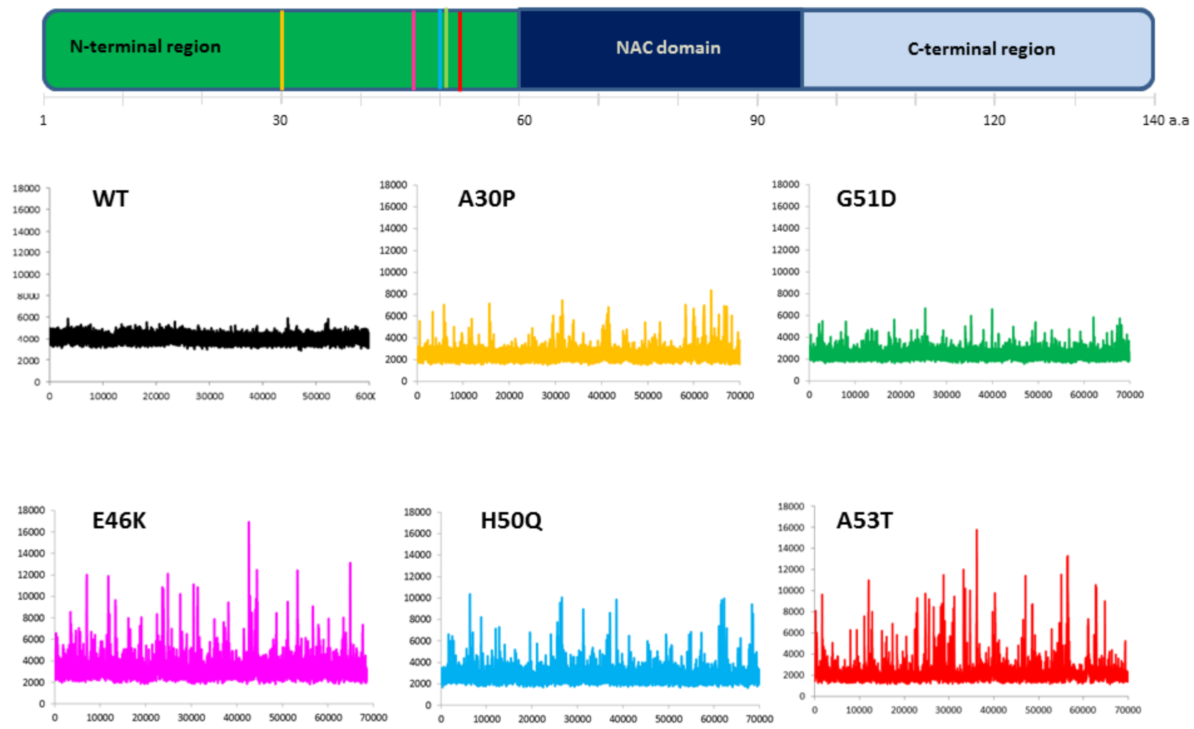

Typical fluorescence traces obtained for WT α-synuclein (black), A30P mutant (yellow), G51D mutant (green), E46K mutant (pink), H50Q mutant (blue) and A53T mutant (red) and relative position of the mutations on a schematic of the synuclein domain composition. C-terminal sGFP-labelled proteins were expressed in 10 µL of LTE using 20 nM of DNA template, incubated for 2h at 27 °C and diluted 10 times in buffer A.

Figure S3- The distribution of intensity values reports on the oligomerization of the proteins.

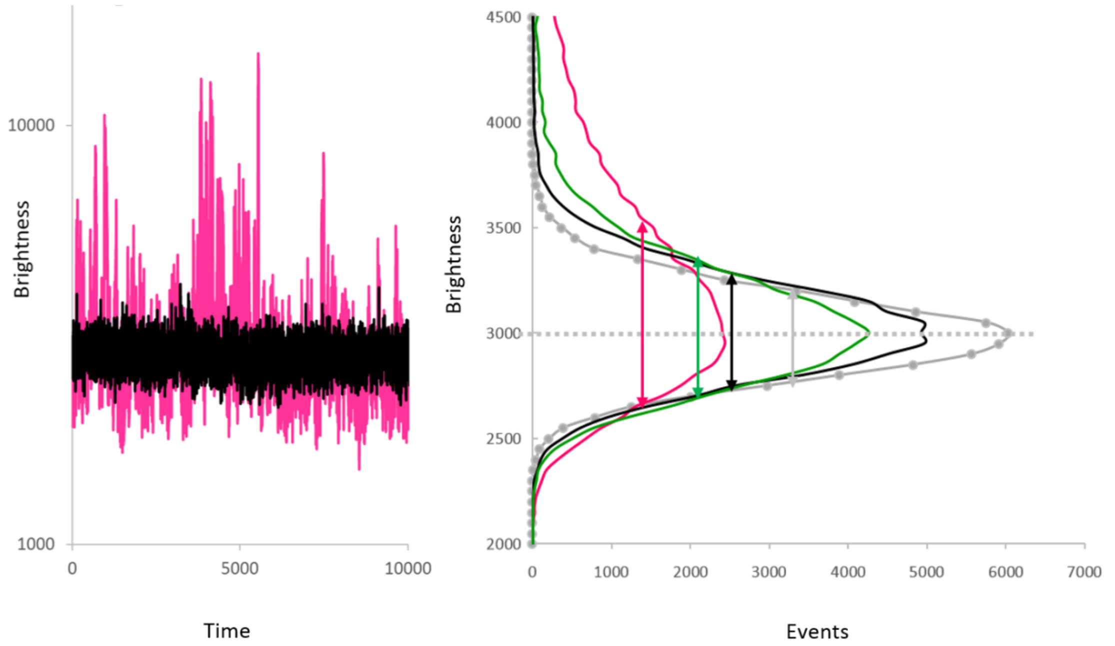

Typical traces for WT α-synuclein (black) and E46K mutant (pink) (left) and brightness analysis for control (sGFP, grey), WT α-synuclein (black), G51D (green) and E46K (pink). When the proteins are expressed at the same level, the means of the distribution align. The presence of large objects is reflected by a widening of the distribution that can be detected and quantified by the standard deviation.

Figure S4- Brightness profile of an inhomogeneous sample

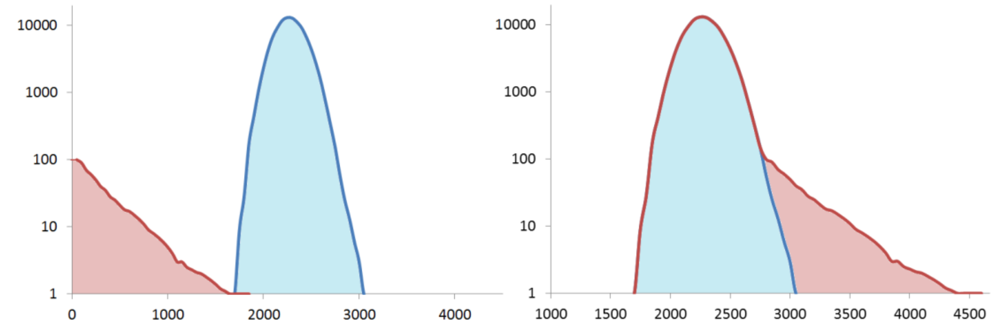

When the proteins form rare large oligomers, the distribution of values becomes largely asymmetric. This distribution can be decomposed into two contributions. The fluctuations created by the dominant species, monomer or small, well-defined oligomer, correspond to the symmetrical Gaussian distribution highlighted in blue. The rare events are typically at single-molecule concentrations so their detection is rare and we obtain a linear distribution of intensities, as shown in red.

Figure S5- The final protein concentration can be tuned precisely by varying the amount of DNA template.

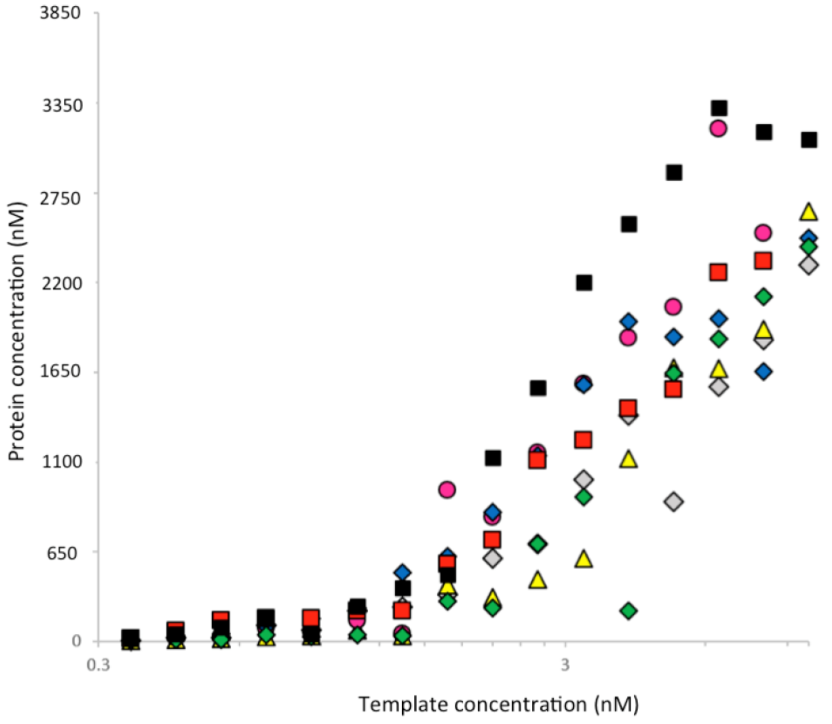

Final protein concentration as a function of DNA template used to initiate translation. Color-coding corresponds to Fig. S2. Different concentrations of DNA are used to prime the translation. GFP fluorescence is measured after 2h of expression at 27 °C. The protein concentrations are calculated from the sGFP fluorescence. A calibration curve was obtained for *E.Coli* purified fluorescent proteins (sGFP and GFP-BBS9) [1].

Figure S6- single-molecule TIRF imaging of A30P and E46K mutants.

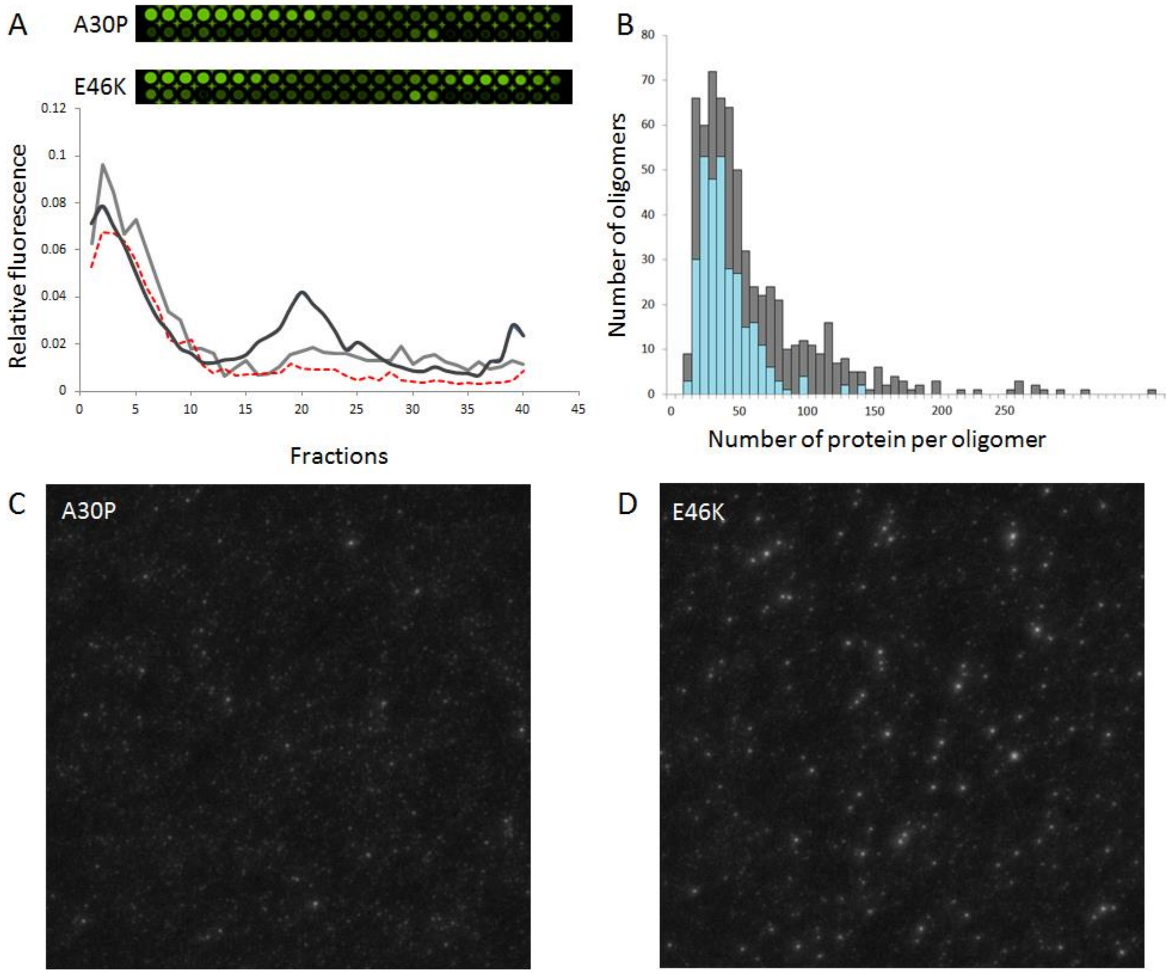

(A) Fractionation of 500 µL of LTE samples that expressed WT α-synuclein, A30P and E46K mutants. After ultracentrifugation on a 10-60% sucrose gradient, the LTE samples were collected as separate fractions in 40 wells of a 384-wells plate. The plate was imaged on a fluorescence gel imaging setup (UVITEC), and the samples were loaded onto the single-molecule fluorescence setup to measure average intensities. For E46K (black), a clear separation was observed between monomeric and aggregated species, with a peak of fluorescence around fraction #20. The profile for A30P (in grey) also shows the presence of oligomers of higher density, detected above the monomer baseline established with WT α-synuclein (red dotted line). Multiple fractions were analysed by single-molecule TIRF (#9, #17, #20 and #25).

(B) Histogram of values obtained by single-molecule TIRF analysis for the oligomeric states of A30P and E46K at fraction #20. The oligomers present for E46K contain more proteins compared to A30P. A30P typically forms oligomers of 30 proteins, while E46K displays a population of aggregates with >100 proteins/aggregate.

(C) Typical single-molecule TIRF image obtained for A30P mutant; a few bright oligomers can be detected and quantified. (D) With the same acquisition parameters, a sample of E46K mutant displays more oligomers with brighter average values.

Figure S7- Co-aggregation of WT and mutants α-synuclein is assessed by two-color single-molecule coincidence.

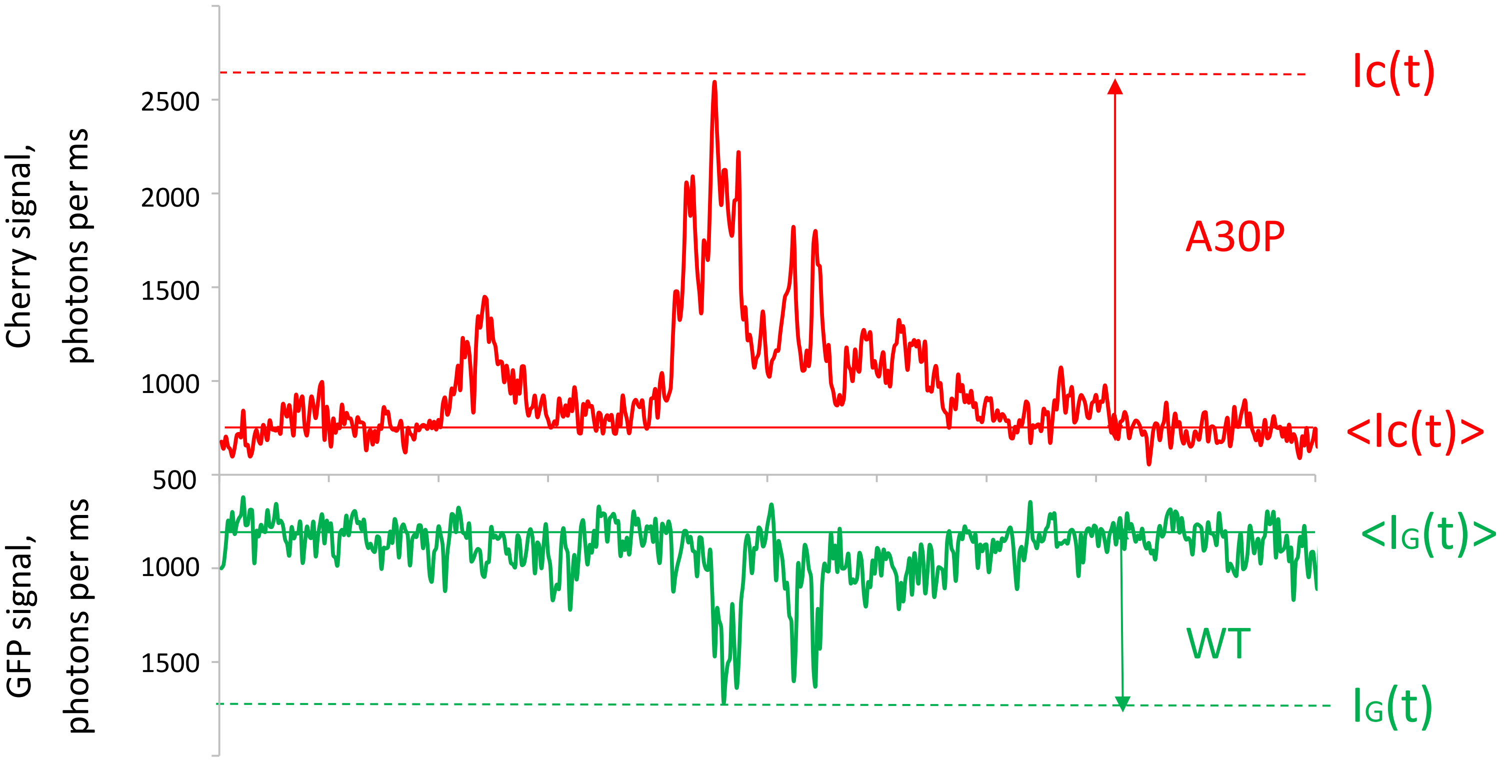

Here WT α-synuclein-sGFP is co-expressed with A30P-mCherry. The trace shows the co-diffusion of the two proteins in a large oligomer. This can be quantified as follows.

The average intensity and standard deviation was calculated for both the Cherry and the GFP traces. For each event in Cherry above threshold (> average of Cherry signal $<I_C(t)>$ + 3 SD), the relative enrichment in GFP over Cherry was calculated as :

$$R = \frac{I_G(t) - < I_G(t) >}{I_C(t) - < I_C(t) >}$$

An event was taken into account if 1) ($< I_C(t) >$ + 3 SD)> ($< I_G(t) >$+ 3 SD) and 2) R<1.

The data presented in Figure 4E are averaged from more than 100 validated events.

Figure S8- The interaction between proteins can be measured by AlphaScreen

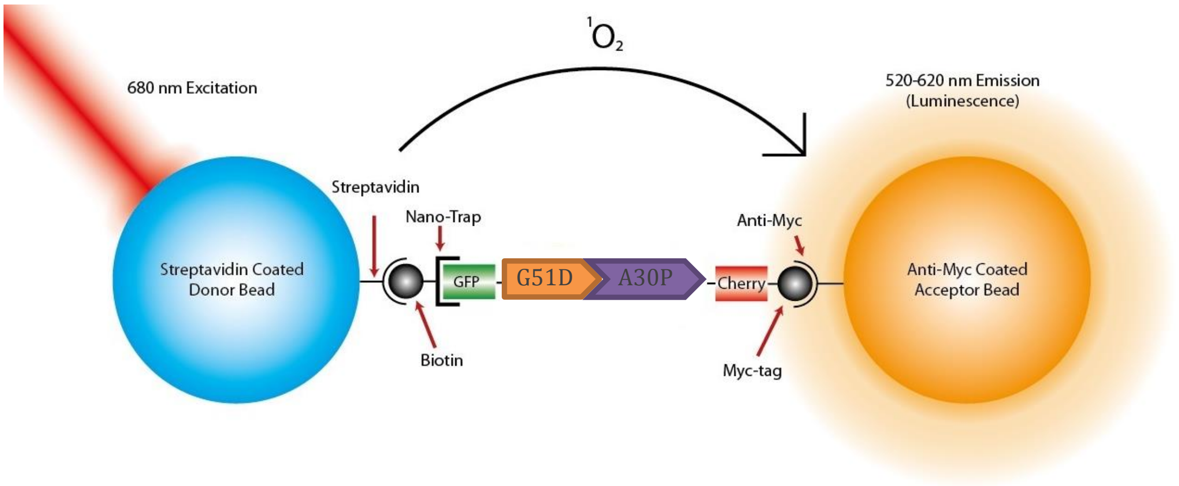

Synuclein G51D labelled with C-terminal GFP is binding to a streptavidin coated donor bead by using a biotinylated GFP-nanotrap. α-Synuclein A30P-mCherry myc is recruited to the acceptor bead by interaction between the Myc tag and the anti-Myc antibody. Interaction between the proteins brings the beads in close proximity. Upon excitation at 680 nm, the donor bead releases a singulet oxygen that can travel in the solution for 200nm before returning to its triplet unexcited state. Reaction between the singulet oxygen and the phtalocyanate contained in the acceptor bead results in emission of light in the 520-620 nm range.

In this study, we are taking advantage of another aspect of the system. We perform serial dilutions of the samples containing the co-expressed proteins to find the optimum of the system. Maximal AlphaScreen signal is detected when we dilute the LTE by 3-4 orders of magnitude. At those dilutions, even in a sample that shows high aggregation in single molecule, the probability of having aggregates is low compared to the number of monomers. Furthermore, the presence of few aggregates should not overwhelm the system and interaction between one aggregate and a monomer should more or less have the same influence than an interaction between two monomers.

Figure S9- The aggregation propensity is blocked by a GFP tag at the N-terminus of α-synuclein

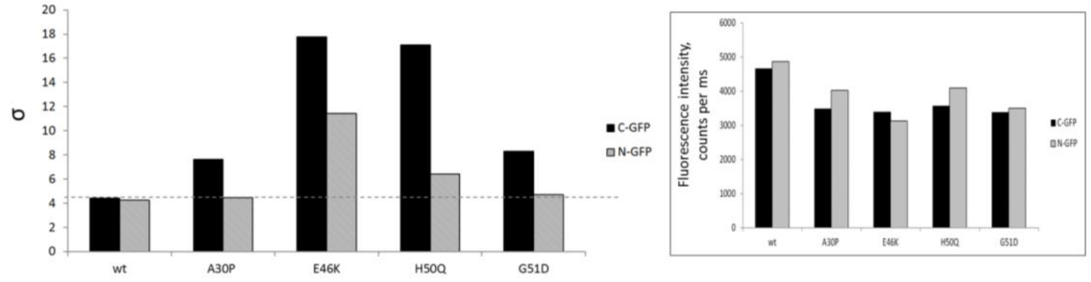

Representative σ values for WT α-synuclein and α-synuclein mutants tagged with sGFP on their N-terminus (black) or C-terminus (dashed). The insert shows the average fluorescence recorded for the selected samples.

Figure S10- The aggregation is not caused by tagging with the fluorescent proteins.

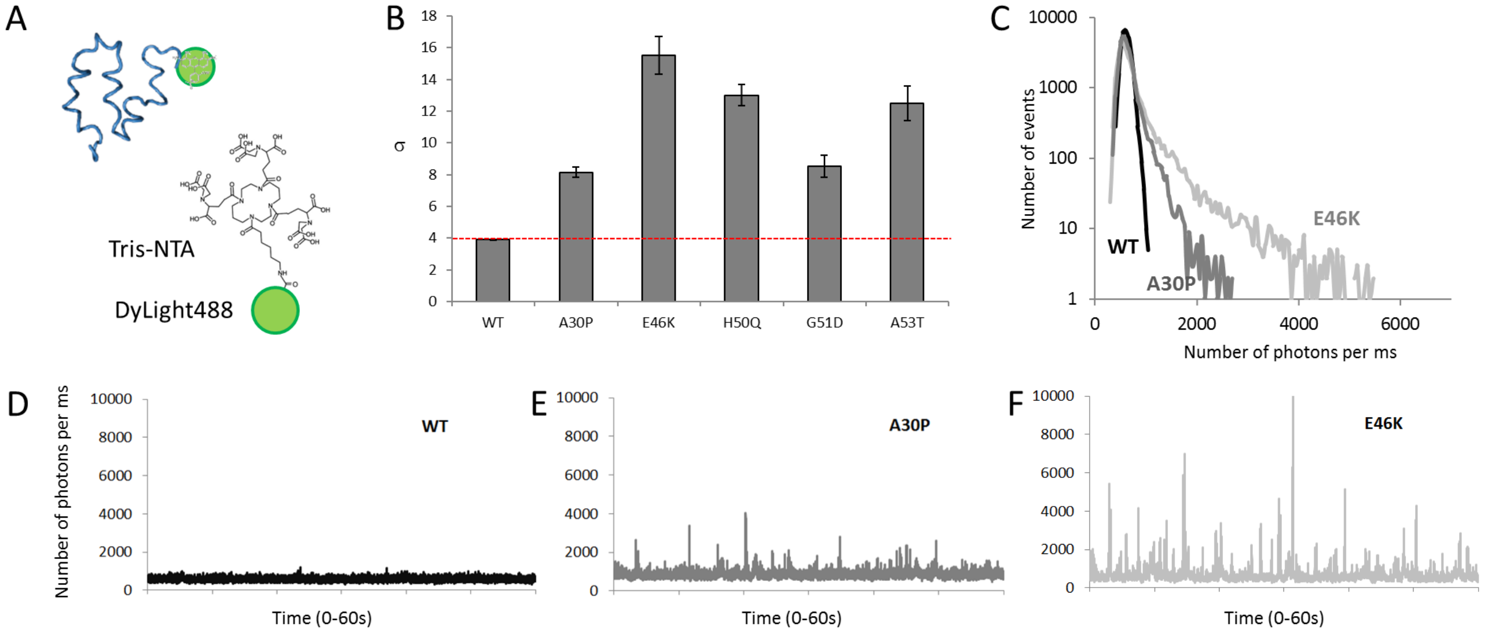

(A) Principle of tagging of unlabelled α-synuclein with a fluorescent derivative of Tris-NTA [2]. The NTA associates tightly with two Histidines residues, and the triple NTA compound has nanomolar affinity for the 6-His tag present on the expressed proteins. (B) Heterogeneity of the time-traces obtained for WT α-synuclein and α-synuclein mutants. The brightness of the DyLight488 is very similar to the brightness of individual sGFP, and the σ values obtained match closely the ones described in Fig.1 and Fig. 2. (C) Typical distribution of intensity values (number of photons per ms) measured for WT α-synuclein, and α-synuclein mutants A30P and E46K. As shown with sGFP, the oligomers measured for A30P and E46K have different characteristics. (D-F) Fluorescence time-traces obtained for WT α-synuclein, and α-synuclein mutants A30P and E46K.

Figure S11- The aggregation propensity is independent of the lysate batches.

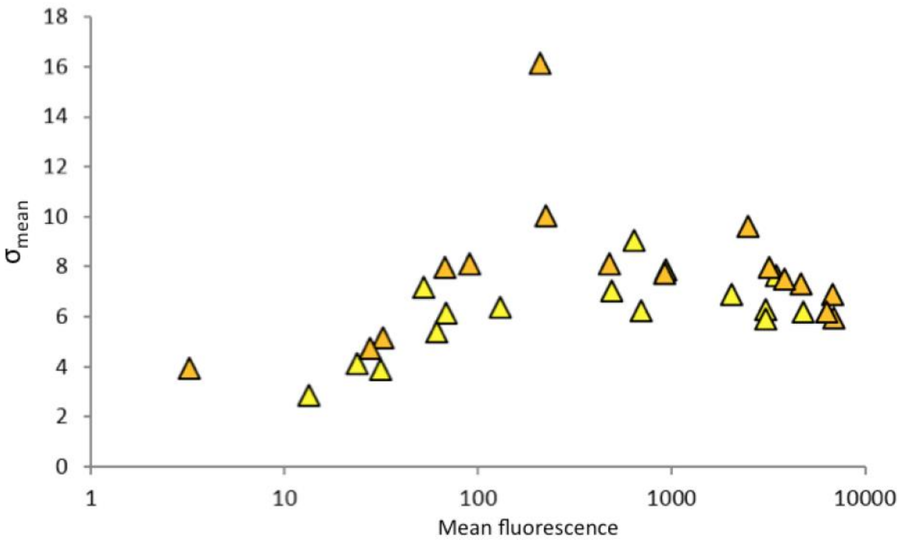

Reproducibility of experiments measuring aggregation as a function of protein expression levels for the A30P α-synuclein mutant. The same experiment was repeated on two different days, with two different batches of cell-free lysate, and with two different serial dilutions of DNA template. The two batches of lysates were chosen to have different lipid and vesicle contents. One lysate (yellow triangles) was manufactured by collecting the top fraction of sample after the last high-velocity spin, to have the lowest amount of lipids and lipid vesicles [3]. The other lysate (orange triangles) followed the classical lysate manufacture protocol. We tested the presence of vesicles by looking at the behaviour of the SNX-BAR proteins. As discussed in a previous work [1] the SNX-BAR family of proteins can bind to lipid vesicles and they oligomerisation status can be used to test the presence of lipid aggregates. The SNX-BAR "reporters" show very different behaviours in the two lysate batches, with clear binding in the typical lysate and monomeric states in the low-lipid lysate.

.

Figure S12- The lysate components do not modify the aggregation behaviour of the proteins

In this study, we used different batches of lysates which are all slightly different in terms of lipid composition, protein content, kinetics of expression… Those parameters depend on the Leishmania cells themselves and as every batch are obtained from a fresh culture, those vary. However, in order to create a cell-free expression system, the Leishmania cell extracts are also supplemented with known components necessary to provide energy, stability or specificity to the translation machinery [3, 4]. To evaluate the effects of some of these additives, we studied the aggregation of α-synuclein WT and mutants A30P and E46K expressed in slightly different conditions. As some elements (ATP, CTP/UTP, antisense oligonucleotides) are essential to the translation machinery, we created lysates with increased amounts of those components. When the components were dispensable for protein expression, we removed them from the mixture, as in the case of the crowding agents PEG and spermidine. Overall, protein expression was only slightly modified (at maximum 1.5x, data not shown) but this effect does not affect the σ_{mean} values, which are concentration-independent parameters.

In all cases, α-synuclein WT and mutants A30P and E46K, tagged with C-terminal GFP, were expressed with a lysate supplemented as described previously (Ctl) or with variable concentrations of different elements. After 3 hours expression, 4 time traces of 30 sec were acquired and the σ_{mean} values were calculated. Data are presented as average σ_{mean} ± SEM. Red bars correspond to α-synuclein WT; dark grey to α-synuclein A30P and light grey to α-synuclein E46K. The overall conclusion is that none of our additives has a pronounced effect on protein oligomerization and most of the aggregation behaviours we observed are not triggered by a specific component of the lysate.

Effect of nucleotide triphosphates

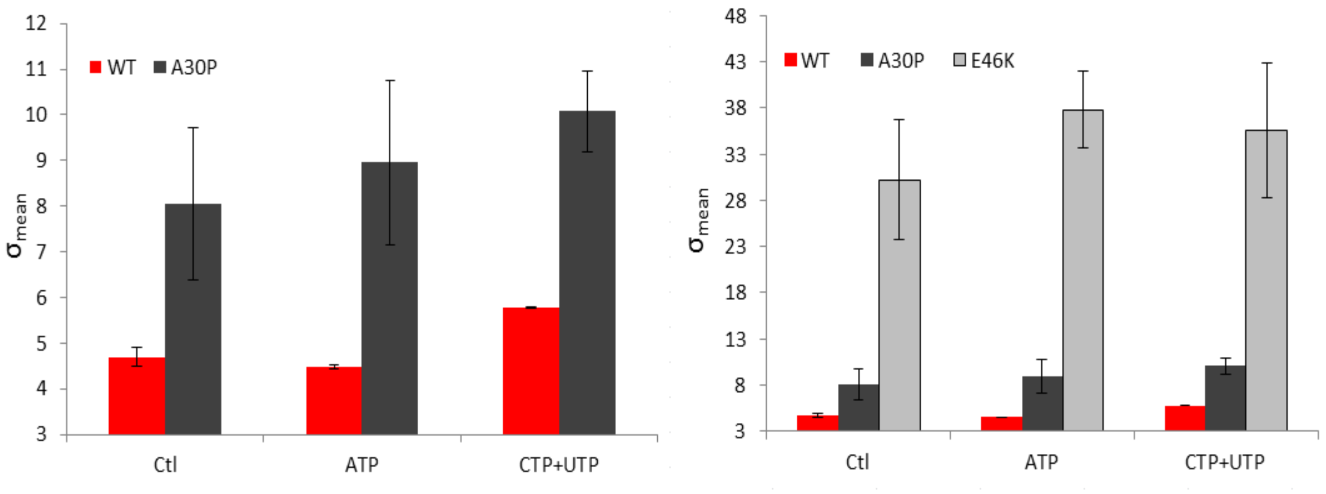

The expression lysate was supplemented as previously described (Ctl, [ATP]= 1.3mM, [CTP]=[UTP]=0.375mM) or with added ATP ([ATP]=6.1mM, [CTP]=[UTP]=0.375mM) or added CTP and UTP ([ATP]=0.375 mM, [CTP]=[UTP]=2.8mM). The average σ_{mean} ± SEM is presented for the different conditions. Additional ATP and CTP/UTP have no significant effect on the aggregation of the different proteins. Increased concentrations of ATP and CTP/UTP block protein expression.

__Effect of oligonucleotides__

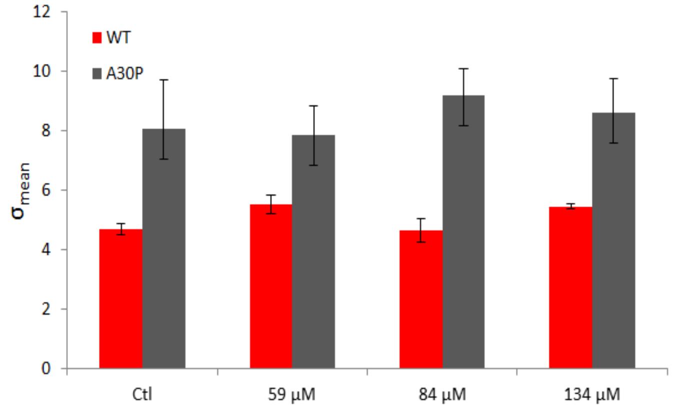

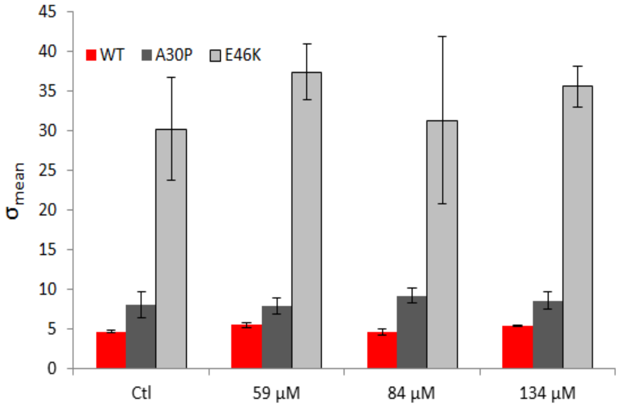

The expression lysate was supplemented as previously described (Ctl) or with increasing amounts of antisense oligonucleotides, to a final concentration of 38 µM in the control and then as indicated. Antisense oligonucleotides are used to prevent translation of endogenous proteins in the lysate. The average σ_{mean} ± SEM is calculated for the different oligonucleotide concentrations. No significant effect on protein aggregation was noticed.

__Effect of crowding agents__

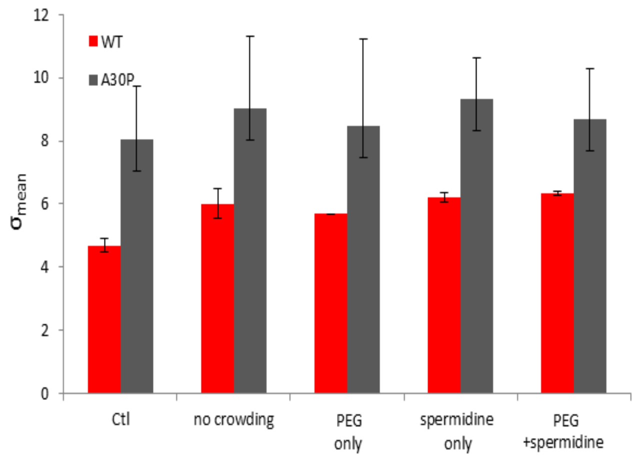

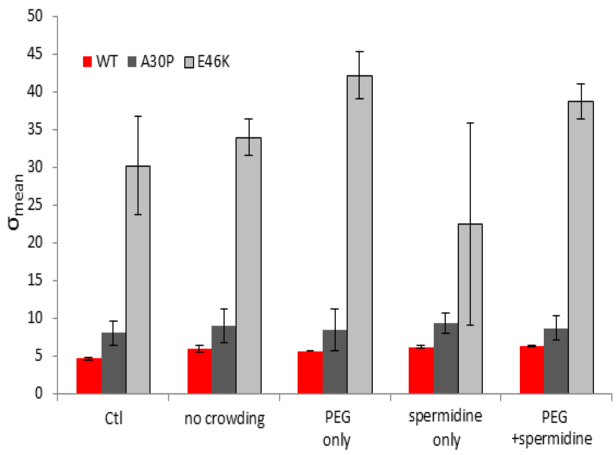

The expression lysate was supplemented as previously described (Ctl, 0.75 %PEG v/v + 0.18mM spermidine), or without PEG or spermidine (no crowding), with 1 % PEG v/v (PEG only) with 1 mM spermidine (spermidine only) or re-supplemented with 1%PEG v/v and 1 mM spermidine (PEG+ spermidine). The average σ_{mean} ± SEM is obtained for the different crowding conditions. Protein aggregation was not significantly modified.

Figure S13- The aggregation process continues over time

Though it is difficult with our technique to evaluate the composition of the different oligomeric species that can be present in the reaction mix, we can estimate the fraction of monomeric protein that remains in the system. To this end, we use the simplifying hypothesis that the monomeric protein is the main contributor to the Gaussian distribution observed in the brightness distribution. We then calculate the number of events (or the area under the curve) in the first half of that Gaussian distribution and multiply by 2 to get the total number of events. The fraction of monomer is then given by the ratio between this number of events and the total number of events recorded in the time trace.

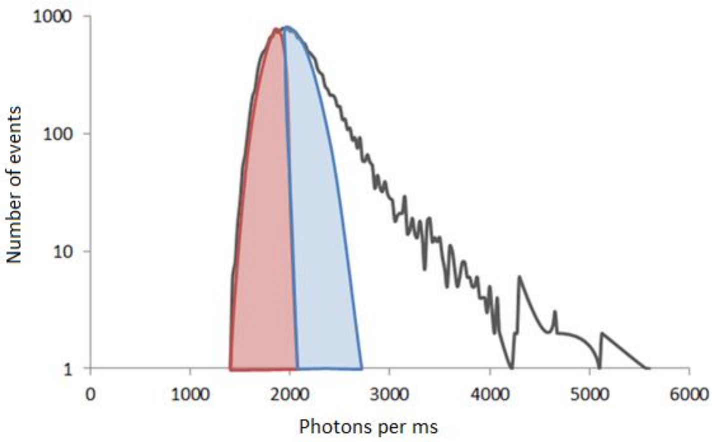

Example of the brightness distribution obtained from a 20sec time trace acquired at 1000Hz for α-synuclein E46K tagged with C-terminal GFP.

The protein was expressed for 3h at 27°C before experiment. The area under half the Gaussian distribution is highlighted in red and encompasses 8,325 events. The whole Gaussian distribution is approximated by doubling this amount (red + blue areas), accounting for 16,650 events. As the time trace contains 20,000 events, the fraction of monomers in solution is approximately 83%. Using the same calculation, we estimate that after 3 h expression at 27°C, α-synuclein WT is mainly monomeric or slightly dimeric (95%) and A30P only forms a small fraction of oligomers (around 7%).

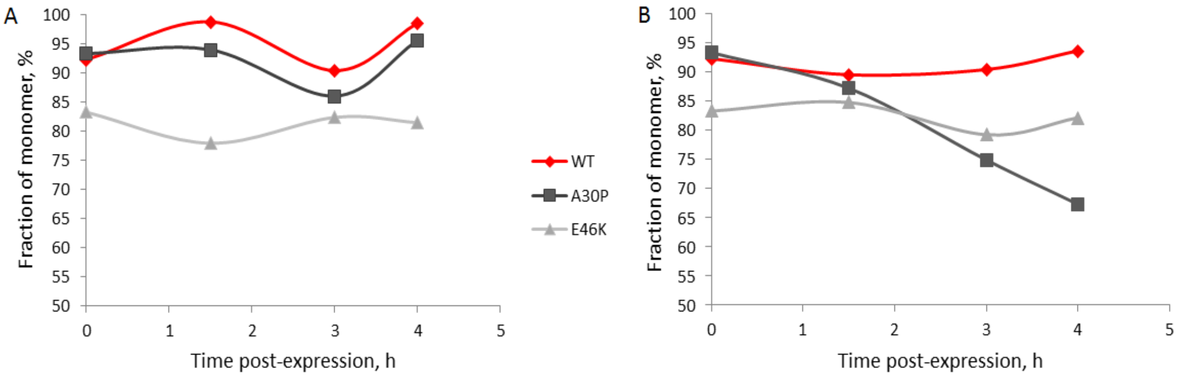

Fraction of monomer in solution over time.

α-Synuclein WT and mutants A30P and E46K, tagged with C-terminal GFP, were expressed for 3h at 27°C as described previously (t=0) then kept at 27°C (A) or 37°C (B) for different amount of time (1.5h, 3h or 4h). For each time point, 4 time traces of 30 sec were acquired and the σ_{mean} values were

calculated. Data are presented as average σ_{mean} ± SEM. Red diamonds correspond to α-synuclein WT; dark grey squares to α-synuclein A30P and light grey triangles to α-synuclein E46K. Incubation at 27°C had no impact on the aggregation profile of the proteins but oligomerization/aggregation increases with time when the samples are kept at 37°C, especially in the case of A30P.

Figure S14- EM imaging shows differences between fibrils of A30P and H50Q

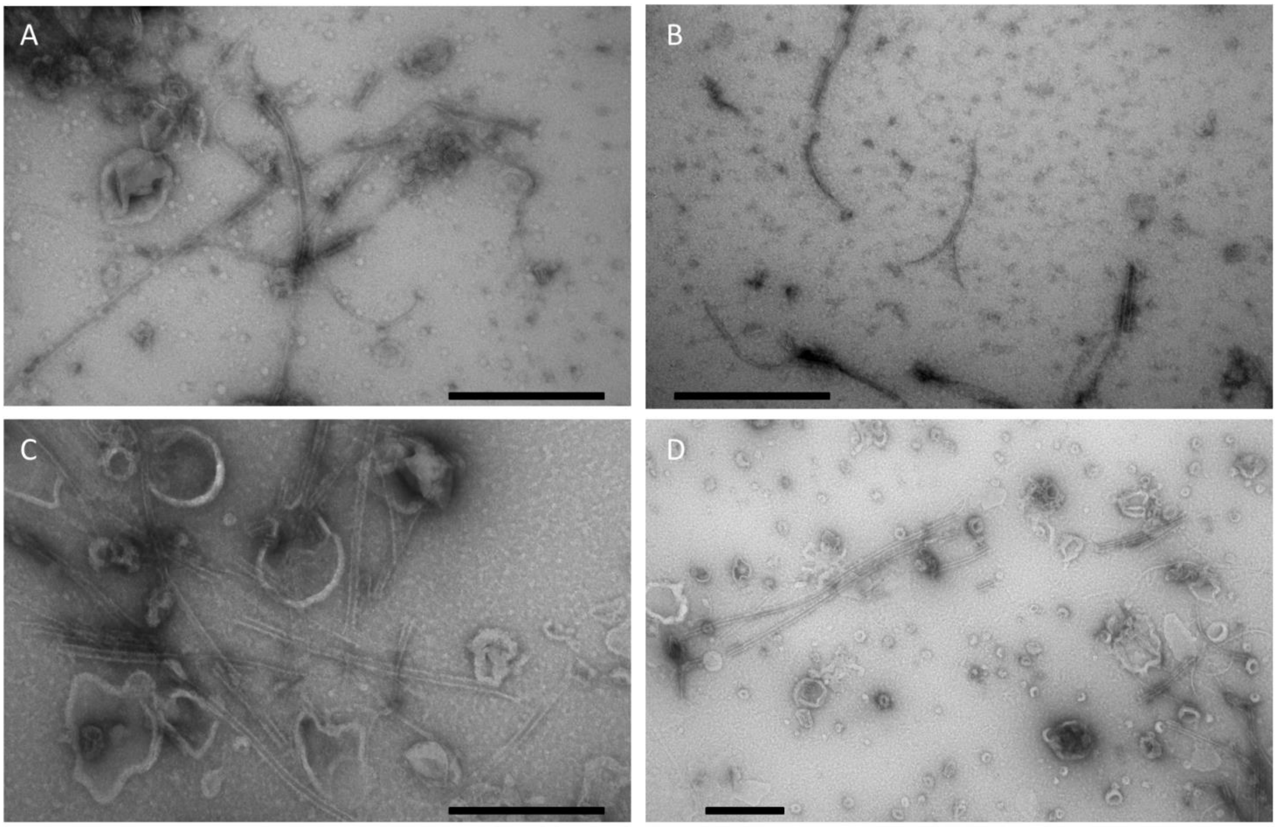

(A-B) Electron Microscopy images of fibrils of A30P obtained as described in Figure 4. (C-D) Electron Microscopy images of fibrils of H50Q obtained in the same conditions. Black bars indicate the scale (500 nm).

The images suggest that the α-synuclein A30P fibrils are smaller and less rigid than the α-synuclein H50Q ones.

Figure S15- Single-particle FRET shows different signatures in the A30P and E46K oligomers.

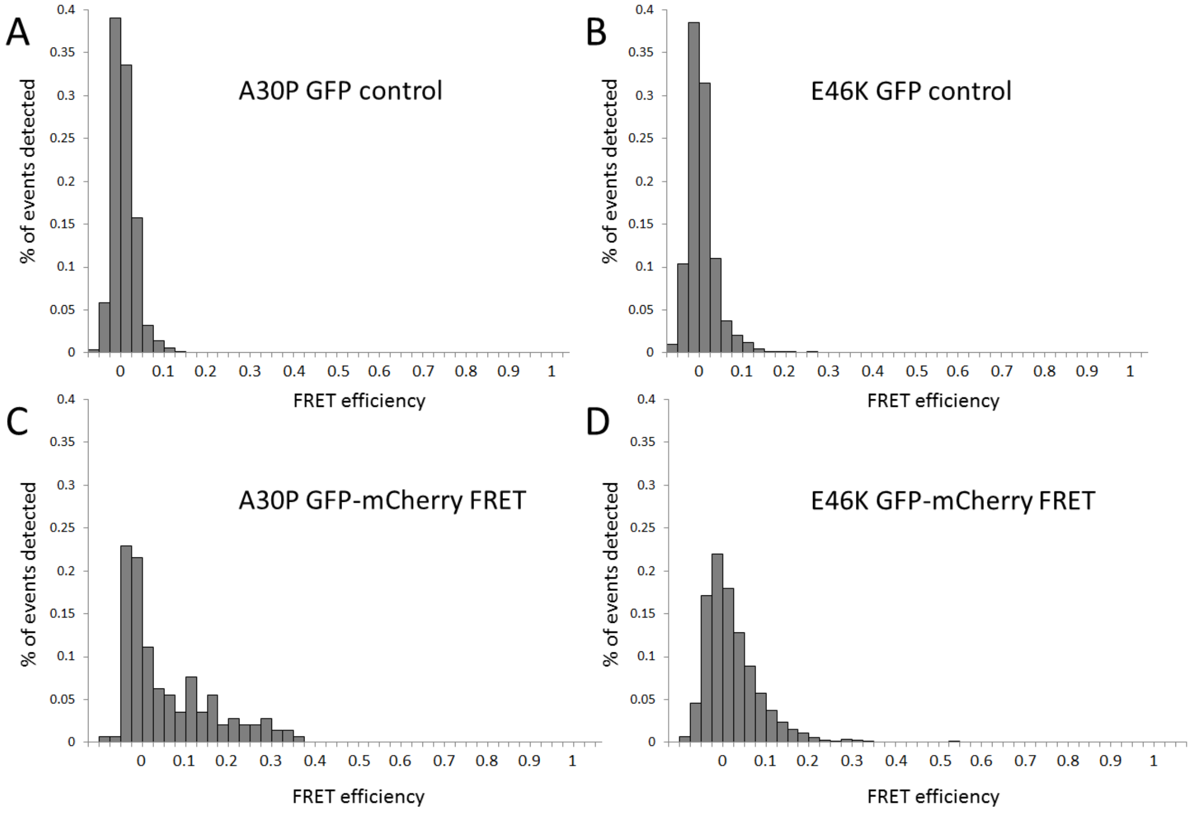

GFP and mCherry tagged versions of the same protein (α-synuclein A30P and α-synuclein E46K) were co-expressed and single-particle FRET was measured on the α-synuclein oligomers.

The GFP-only samples (A,B) were used to check that the background and leakage between detection channels are corrected accurately. (C, D) A single excitation laser (488nm) was used and the donor fluorescence (GFP) and the FRET-driven acceptor fluorescence (mCherry) were measured separately. The FRET efficiency was then measured as the corrected Cherry signal (I_C), divided by the total intensity of the burst (= $I_C/[I_G+I_C]$). Histograms of percentage of events as a function of FRET efficiency were obtained by measuring >100 separate oligomers.

The results show the presence of FRET but to limited values, E_{FRET}= 0.2 for A30P and E_{FRET}< 0.1 for E46K. The difference in FRET profiles confirms that the two types of oligomers are different.

REFERENCES

1. Sierecki, E. *et al.* Rapid mapping of interactions between human SNX-BAR proteins measured in vitro by AlphaScreen and single-molecule spectroscopy. *Molecular & cellular proteomics : MCP*, doi:10.1074/mcp.M1113.037275 (2014).
2. Lata, S., Gavutis, M., Tampé, R. & Piehler, J. Specific and Stable Fluorescence Labeling of Histidine-Tagged Proteins for Dissecting Multi-Protein Complex Formation. *Journal of the American Chemical Society* **128**, 2365-2372 (2006).
3. Mureev, S., Kovtun, O., Nguyen, U.T. & Alexandrov, K. Species-independent translational leaders facilitate cell-free expression. *Nat Biotechnol* **27**, 747-752 (2009).
4. Kovtun, O. *et al.* Leishmania cell-free protein expression system. *Methods* **55**, 58-64 (2011).
